# A novel multiplex assay for simultaneous quantification of total and S129 phosphorylated human alpha-synuclein

**DOI:** 10.1186/s13024-016-0125-0

**Published:** 2016-08-22

**Authors:** Natalie Landeck, Hélène Hall, Mustafa T. Ardah, Nour K. Majbour, Omar M. A. El-Agnaf, Glenda Halliday, Deniz Kirik

**Affiliations:** 1 Brain Repair and Imaging in Neural Systems, Department of Experimental Medical Science, Lund University, BMC D11, 22184 Lund, Sweden; 2 Department of Biochemistry, College of Medicine and Health Sciences, United Arab Emirates University, Al Ain, United Arab Emirates; 3 Neurological Disorders Research Center, Qatar Biomedical Research Institute (QBRI), Education City, Qatar Foundation, P.O. Box 5825, Doha, Qatar; 4 College of Science and Engineering, Hamad Bin Khalifa University (HBKU), Education City, Qatar Foundation, P.O. Box 5825, Doha, Qatar; 5 Faculty of Medicine, University of New South Wales and Neuroscience Research Australia, 2052 Sydney, Australia; 6 Current address: Department of Pharmacology and Therapeutics, McGill University, Montréal, Canada

**Keywords:** Alpha-synuclein, AlphaLISA, Multiplex assay, Phosphorylated alpha-synuclein, Synucleinopathy

## Abstract

**Background:**

Alpha-synuclein (asyn) has been shown to play an important role in the neuropathology of Parkinson’s disease (PD). In the diseased brain, classic intraneuronal inclusions called Lewy bodies contain abnormal formations of asyn protein which is mostly phosphorylated at serine 129 (pS129 asyn). This suggests that post-translational modifications may play a role in the pathogenic process. To date, several uniplex assays have been developed in order to quantify asyn not only in the brain but also in cerebrospinal fluid and blood samples in order to correlate asyn levels to disease severity and progression. Notably, only four assays have been established to measure pS129 asyn specifically and none provide simultaneous readout of the total and pS129 species. Therefore, we developed a sensitive high-throughput duplex assay quantifying total and pS129 human asyn (h-asyn) in the same well hence improving accuracy as well as saving time, consumables and samples.

**Results:**

Using our newly established duplex assay we measured total and pS129 h-asyn in vitro showing that polo-like kinase 2 (PLK2) can phosphorylate asyn up to 41 % in HEK293 cells and in vivo the same kinase phosphorylated h-asyn up to 17 % in rat ventral midbrain neurons. Interestingly, no increase in phosphorylation was observed when PLK2 and h-asyn were co-expressed in rat striatal neurons. Furthermore, using this assay we investigated h-asyn levels in brain tissue samples from patients with PD as well as PD dementia and found significant differences in pS129 h-asyn levels not only between disease tissue and healthy control samples but also between the two distinct disease states especially in hippocampal tissue samples.

**Conclusions:**

These results demonstrate that our duplex assay for simultaneous quantification is a useful tool to study h-asyn phosphorylation events in biospecimens and will be helpful in studies investigating the precise causative link between post-translational modification of h-asyn and PD pathology.

**Electronic supplementary material:**

The online version of this article (doi:10.1186/s13024-016-0125-0) contains supplementary material, which is available to authorized users.

## Background

Parkinson’s disease (PD) is the second most common neurodegenerative disorder affecting 1 % of people over the age of 60 [1, 2]. Motor symptoms appear when 30–50 % of dopaminergic neurons in the substantia nigra (SN) and 70–80 % of dopaminergic terminals in the striatum are lost [3, 4]. The cascade of pathological events underlying the neurodegenerative process are unknown, although a number of mechanisms such as impaired protein clearance, oxidative stress, mitochondrial defects and neuroinflammation have been suggested to play a role [5–8]. The surviving neurons in the SN display intracellular inclusions called Lewy bodies (LBs). In fact, these inclusions are also found in cortex, amygdala, locus coeruleus, olfactory bulb and the peripheral autonomic system of patients suffering from PD [9, 10].

LBs were consistently found to contain a large amount of accumulated alpha-synuclein (asyn) [11]. Analysis of abnormally processed and aggregated asyn from patients have revealed different post-translational modifications, including phosphorylation, nitration, ubiquitination and C-terminal truncation of the protein [12–14]. Most of the asyn accumulated in brains of patients is indeed phosphorylated especially at serine 129 (pS129) [14, 15]. These findings lead to the development of independent assays monitoring total asyn levels as well as its oligomeric and phosphorylated states, not only in brain tissue but also in cerebrospinal fluid (CSF) and blood samples [16–22]. Although assays for asyn as a wet biomarker have been developed, particularly to quantify total asyn levels for correlation with disease onset and progression, only five assays have been developed for quantifying pS129 asyn [15, 22–25]. Notably, none of the above methods implemented a duplex assay platform to measure the total asyn and pS129 asyn in a single well. This is particularly important not only to save samples, consumables and time but also to measure asyn phosphorylation ratios more accurately especially when pS129 asyn levels are very low.

Here, we describe a novel assay which allows simultaneous, sensitive and specific analysis of total and pS129 human asyn (h-asyn) species directly in the same well. The assay provides a wide dynamic range permitting a single run even when there are highly varying h-asyn loads between samples. We provide validation by successfully measuring total and pS129 h-asyn levels simultaneously in human cell culture lysates, as well as in human and humanized rat brain lysates.

## Results

### Specificity of antibodies for establishing human alpha-synuclein AlphaLISA assays

**Fig. 1:**
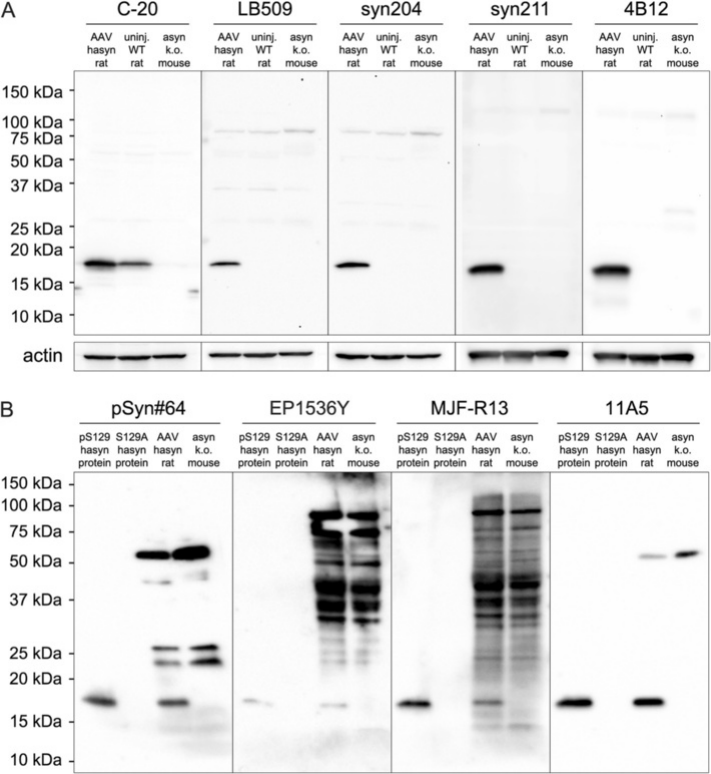
Evaluation of antibodies in relation to species specificity and S129 phosphorylation state*.* The species specificity of asyn antibodies used in the assay development (see Table 1) was tested using Western blot analysis by loading three different lysates – rat brain samples overexpressing h-asyn (AAV hasyn rat), uninjected wild-type rats (uninj. WT rat) or asyn knockout mouse brain tissue (asyn k.o. mouse) (**a**). For lysates 50 μg of protein was loaded. Actin is shown as a loading control. The specificity of antibodies for pS129 state was assessed using recombinant pS129 h-asyn protein or S129A h-asyn protein. Two additional lanes were loaded with either rat brain lysate overexpressing h-asyn (AAV hasyn rat) or asyn knockout mouse brain lysate (asyn k.o. mouse) to evaluate unspecific binding of antibodies on actual samples (**b**). For lysates 100 μg of protein per lane and for recombinant h-asyn 2 ng of protein were loaded

We began our work towards developing an asyn selective assay by examining the specificity of various commercially available or custom generated antibodies to asyn (Table 1). The initial evaluations were performed using WB of tissue obtained from rats overexpressing h-asyn, uninjected wild-type rats, or mouse tissue from asyn knockout animals (Fig. 1a). Whereas the C-20 antibody (Santa Cruz, USA) recognized both the rat and human asyn protein at the expected molecular weight of 17 kDa, LB509 (Covance, USA), syn204 (Cell Signaling, USA), syn211 (Abcam, UK) and 4B12 (Covance, USA) antibodies appeared to be specific to the human variant. None of the five antibodies tested here showed strong binding to another target as documented by the absence of any strong bands on the respective lanes loaded with asyn knockout tissue.

**Table 1:** Antibodies (at 1 mg/ml) used for establishing AlphaLISA assays are listed below together with provider information

Clone	Company	Catalog #	Host species	Clonality	Epitope	Specific detection of		
						Human	Rodent	S129 phos
C-20	Santa Cruz	sc-7011-RL	rabbit	polyclonal	C-terminus	yes	yes	no
LB509	Covance	SIG-39725	mouse	monoclonal	115–122	yes	no	no
asyn204	Cell Signaling	2647BF	mouse	monoclonal	087–111	yes	no	no
asyn211	Abcam	ab80627	mouse	monoclonal	121–125	yes	no	no
4B12	Covance	SIG-39730	mouse	monoclonal	103–108	yes	no	no
pSyn#64	WAKO	014-20281	mouse	monoclonal	124–134	yes	yes	yes
EP1536Y	Abcam	ab173323	rabbit	monoclonal		yes	yes	yes
MJF-R13	Abcam	ab176839	rabbit	monoclonal		yes	yes	yes
11A5	Prothena	N/A	mouse	monoclonal	124–134	yes	yes	yes

Next, we investigated the specificity of four antibodies to pS129 asyn and any reactivity to the non-phosphorylated variant. All antibodies tested at this step, namely pSyn#64 (WAKO, Japan; note that this antibody has since been discontinued), EP1536Y (Abcam, UK), MJF-R13 (Abcam, UK) and 11A5 (Kind gift from Prothena Biosciences Inc.) showed an expected band at approximately 17 kDa in the pS129 h-asyn recombinant protein lane but lacked a corresponding band for the S129A h-asyn recombinant protein, a non-phosphorylatable asyn variant (Fig. 1b). Different from the case above, two of the four antibodies tested here (EP1536Y and MJF-R13) resulted in several strong bands in the knockout tissue suggesting cross-reactivity to other protein species. Only the custom synthesized 11A5 antibody had a favorable outcome with a strong specific reactivity towards the pS129 asyn and only a single non-specific band visible in the lane loaded with mouse knockout tissue (Fig. 1b).

### Selection of antibody pairs for the total and pS129 human alpha-synuclein specific AlphaLISA assays

The results above prompted us to take the next step in the development of the AlphaLISA assay targeting the h-asyn protein. For this purpose, we first tested the signal to background (S/B) ratio for pairs of antibodies towards detection of either total h-asyn or the pS129 h-asyn protein (Table 2; section to the left). Four human specific antibodies tested in the WB experiment were included in the total h-asyn assay development step. The results suggested that the 4B12 antibody worked best when conjugated to biotin and tested against the syn211 and LB509 antibodies coupled to Europium Acceptor-beads (bold figures in Table 2). Notably, pairing the same antibodies in the opposite orientation did not give the same robust readout but were 2–5 fold lower in signal-to-background ratio. Furthermore, as epitopes used to raise the 4B12 and syn 204 antibodies have overlapping amino acid residues (especially at position 103 and 108; see Table 1), when these two antibodies were paired, there was no detectable signal. Similarly, the epitope for LB509 and syn211 antibodies share amino acid residues 121 and 122, which are in fact different between the rodent and human asyn protein. We have not tested this combination as we anticipated the same outcome as above. Next, we tested the three antibodies specific for pS129 asyn after they were conjugated to the Europium Acceptor-beads and paired with biotinylated 4B12 and syn204 (Table 2; section to the right). This test revealed that the 4B12 – 11A5 antibody combination was far superior (more than 6-fold higher signal-to-background ratio) to other pairs and thus was selected for further development.

**Table 2:** Antibody sensitivity in total (left) and pS129 h-asyn (right) assay is shown as signal to background ratios for each pair

		AB’s conjugated to Europium Acceptor-beads (50 μg/ml)							
		LB509	syn211	syn204	4B12		EP1536Y	MJF-R13	11A5
Biotinylated AB’s (5nM)	LB509	0.7	N/A	16.4	84.8		N/A	N/A	N/A
	syn211	N/A	2.5	9.2	233.5		N/A	N/A	N/A
	syn204	174.1	100.2	1.0	1.1		15.3	101.8	21.2
	4B12	**440.9**	**576.3**	1.1	0.9		35.8	114.6	**704.2**

*N/A* not analyzed

Bold data signifies most favorable signal to background ratios

### Optimization of the AlphaLISA assay condition for uniplex detection of total human alpha-synuclein or pS129 human alpha-synuclein

**Fig. 2:**
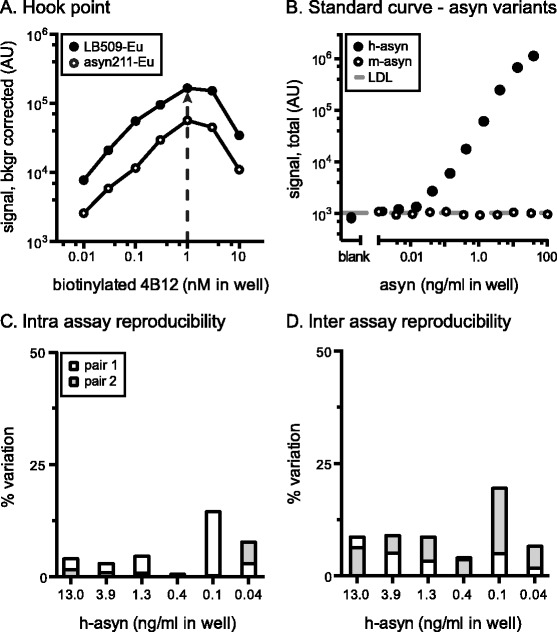
Characterization of total human alpha-synuclein AlphaLISA. The hook-point analysis was evaluated for serial dilutions of biotinylated 4B12 in combination with Europium Acceptor-beads coupled to either LB509 (LB509-Eu) or syn211 (syn211-Eu) using a 19 ng/ml of recombinant GST-tagged h-asyn protein diluted in 10 μg/ml of Tris wild-type rat brain lysate (**a**). *Arrow* indicates the highest signal obtained and therefore optimal 4B12-biotin concentration to use. Standard curve was established for biotinylated 4B12 and Europium Acceptor-bead coupled LB509 by serial dilution of either GST-tagged h-asyn or mouse asyn (m-asyn) protein spiked in Tris brain lysate obtained from a naïve rat (10 μg/ml) (**b**). The lower detection limit (LDL) was 3.7 pg/ml and is indicated by a *dashed line*. Intra assay variation was calculated based on two standard curves which were measured on the same day but on separate plates (**c**). Inter assay variation data was calculated based on two pairs of standard curves performed on separate days (**d**). *AU* arbitrary units

The next step after selection of antibody pairs is the hook-point evaluation, which refers to the optimal concentration of the biotinylated antibody in the assay. In the case of the total h-asyn assay, we performed this analysis for both the LB509 and syn211 antibodies conjugated to Acceptor-beads using the biotinylated 4B12 antibody at concentrations between 10 pM and 10 nM (Fig. 2a). We found that at a concentration of 1.0 nM of biotinylated 4B12 both antibody pairs reached their maximum signal. As the signal from biotinylated 4B12 and LB509 coupled Europium Acceptor-bead pair was three times higher at all concentrations tested we selected this pair as the best condition for detecting total h-asyn.

To evaluate the dose-response characteristics for the total h-asyn AlphaLISA assay, a standard curve was established using either recombinant GST-tagged h-asyn or recombinant mouse asyn (m-asyn) protein spiked into wild-type rat brain lysate (Fig. 2b). The standard curve for h-asyn displayed a typical sigmoid curve, while the signal for m-asyn did not exceed the lower detection limit (LDL). We identified that the linear dynamic range of the h-asyn specific assay lies between 0.1 and 10 ng/ml in-well concentration, while the estimated detection limit is about 2.0 pg/ml and indicates a 3-times higher sensitivity than what previous asyn assays have reported [18–20]. Raw background counts from PBS solution (106.0 ± 11.3) was indifferent from rodent brain lysate readings (121.2 ± 16.9) demonstrating that the total asyn assay has essentially no unspecific signal. Intra and inter assay variations were calculated between four individual standard curves using six concentrations on those curves to validate the accuracy and variation between assay runs (Fig. 2c, d) and 22 out of 24 measurements were below 8.7 %, with two measurements (0.1 ng/ml) at 14.5 and 19.5 % (Fig. 2c, d).

**Fig. 3:**
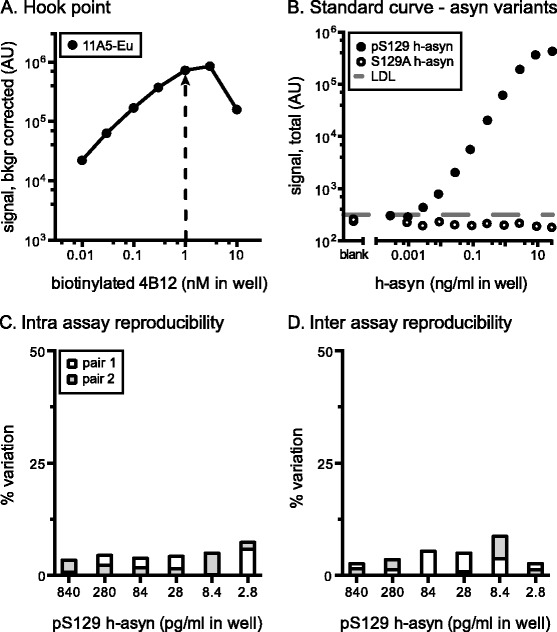
Characterization of S129 phosphorylated human alpha-synuclein specific AlphaLISA. The hook-point analysis was evaluated for biotinylated 4B12 and Europium Acceptor-bead conjugated 11A5 (**a**). Pair was tested using 14 ng/ml of pS129 h-asyn protein diluted in 10 μg/ml of Tris brain lysate obtained from an asyn knockout mouse. Arrow indicates the optimal biotinylated antibody concentration to use. Standard curve was established for biotinylated 4B12 and Europium Acceptor-bead coupled 11A5 by serial dilution of either pS129 h-asyn or S129A h-asyn protein spiked in wild-type rodent brain lysate (10 μg/ml) (**b**). The lower detection limit (LDL) was 1.1 pg/ml and is indicated by a dashed line. Intra assay variation was calculated based on two standard curves which were measured on the same day but on separate plates (**c**) while the inter assay variation was calculated based on two pairs of standard curves performed on separate days (**d**). *AU* arbitrary units

The hook-point analysis for the pS129 h-asyn AlphaLISA assay showed that, while 3.0 nM of biotinylated antibody provided the highest signal for the 4B12 – 11A5 antibody pair (Fig. 3a), using 1.0 nM of antibody resulted in only 17.4 % of signal drop while saving 3-times the amount of antibody. Dose-response characteristics of the pS129 h-asyn assay was analyzed by establishing standard curves using pS129 h-asyn and S129A h-asyn recombinant protein spiked into asyn knockout mouse brain lysate (Fig. 3b). The standard curve for pS129 h-asyn displayed a typical sigmoidal shape while signal for the S129A h-asyn between 1 pg/ml and 100 ng/ml concentrations remained below the LDL. To ensure accurate calculations for sensitivity and linear dynamic range of the pS129 h-asyn assay, we measured the amount of phosphorylated h-asyn using isoelectric focusing gels and determined 28 % of our recombinant protein standard to be phosphorylated (Additional file 1: Figure S1). All values reported for pS129 h-asyn have been adjusted using this factor. The linear dynamic range of the pS129 h-asyn assay for the pS129 h-asyn protein is between 8 pg/ml and 3 ng/ml in-well concentration with an LDL of 0.3 pg/ml, which is about 30-times lower than a previously described pS129 asyn assay [24]. Background signal for PBS solution (93.8 ± 10.8) was not distinguishable from rodent brain lysate readings (114.9 ± 34.6) showing that the pS129 h-asyn assay is free from interfering non-specific signal. Intra and inter assay variations were again calculated using six different concentrations on four individually run standard curves and was lower than 8.7 % for all measurements (Fig. 3c, d).

### Feasibility and performance assessment of duplex AlphaLISA assay for quantification of total and pS129 human alpha-synuclein in a single well

The challenge in designing a duplex detection method in an antibody based quantitative assay for a single protein target in two confirmations, as opposed to one where two separate protein targets are measured in the same well, is two-fold: First, a fraction of the target protein is expected to be labeled with two antibodies linked to Acceptor-beads and a third antibody-biotin complex to anchor the Donor-bead to the same target (Additional file 2: Figure S2). Thus, the assay validation steps have to include assessment of steric hindrance at the binding site of either Acceptor-bead coupled antibody by the other. Second, as the two Acceptor (reporter) beads rely on the same Donor (activator) bead but only one of them is read at a time, the sequence of acquisition of the signal needs to be considered.

**Fig. 4:**
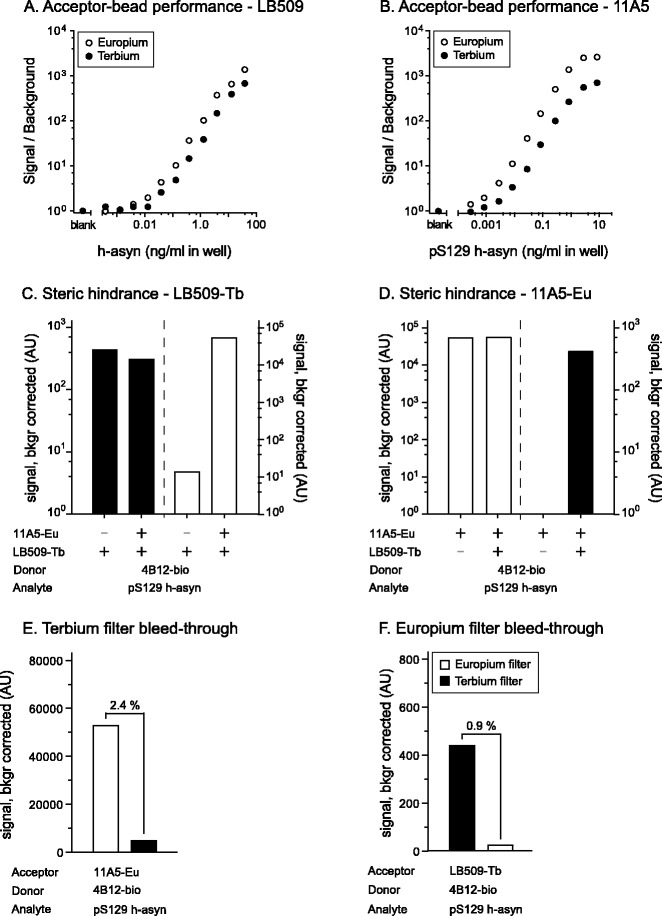
Evaluation of the duplex AlphaLISA performance. Assessment of the Acceptor-bead performance and comparison between Europium and Terbium based beads was carried out once for both the LB509 (**a**) and the 11A5 (**b**) antibodies. In both instances the biotinylated 4B12 antibody was used and signal to background ratio calculated against serial dilutions of h-asyn proteins spiked in naïve rat brain lysate. Presence of any hindrance of Acceptor-bead coupled antibodies against each other was assessed for LB509-Terbium (**c**) and 11A5-Europium beads (**d**). Cross-talk between the channels was assessed using the Resorufine/Amplex Red FP535 Terbium filter (**e**) and the Europium 615 filter (**f**). For these experiments the relevant analyte, Acceptor- and Donor-bead information is given under the x-axis. Both the hindrance of Acceptor-bead coupled antibodies and the channel cross talk were run twice. See Additional file 2: Figure S2 for a theoretical explanation of the phenomena. *AU* arbitrary units

To this end, our first task in designing the duplex assay for h-asyn and its post-translationally modified pS129 species was to evaluate the characteristics of the Terbium Acceptor-beads compared to Europium Acceptor-beads for the LB509 and 11A5 antibodies. Signal characteristics of both Acceptor-beads were tested by combining biotinylated 4B12 antibody with LB509 antibody coupled to either Europium or Terbium Acceptor-beads separately and by using purified protein in a dose-response curve. Overall Terbium signal was about 100-fold lower in all measurements including blanks as compared with the Europium signal under identical conditions. As the background signal in this assay was also significantly lower, the S/B ratio of the standard curves obtained using Terbium beads were only marginally affected as compared with the Europium Acceptor-beads (Fig. 4a). The same setup was used for the 4B12 and 11A5 antibody pair (Fig. 4b). In the latter case the difference in S/B between Europium and Terbium Acceptor-beads was more pronounced. Because of this phenomenon and the fact that there will always be a higher quantity of total h-asyn compared to pS129 h-asyn, we chose to use Terbium Acceptor-beads coupled to LB509 and conjugated the 11A5 antibody to Europium Acceptor-beads. We concluded also that reading the Terbium signal first followed by the Europium signal next was the best protocol.

The epitopes of the two Acceptor-bead coupled antibodies in our assay were very close (see Table 1 for details). Therefore, we explored whether the steric hindrance affected the results by comparing the signal intensity of uniplex and duplex condition for each Acceptor-bead coupled antibody (see Additional file 3: Figure S3 for graphical illustration of the principle). When the signal is the same for both conditions, no hindrance occurs when both antibodies are binding to the analyte (Additional file 3: Figure S3). When the signal in the duplex condition is lower than in the uniplex condition, the other Acceptor-bead coupled antibody complex is sterically hindering the binding (Additional file 3: Figure S3). We found that the signal obtained from Terbium Acceptor-bead coupled LB509 (LB509-Tb) [Fig. 4c; where signal detected from the Terbium filter (Resorufine/Amplex Red FP535) for both conditions is displayed on the left, while the signal detected *via* the Europium filter (Europium 615 nm) of the same wells are shown on the right] was not affected due to presence of the 11A5-coupled Europium Acceptor-bead (11A5-Eu). Similarly, the signal obtained from the 11A5-Eu Acceptor-beads were not influenced by the addition of Acceptor-beads coupled to the LB509 antibody (Fig. 4d).

Next, to ensure that the signals measured for Terbium and Europium were indeed not influenced by one another, we tested their respective filters for signal bleed-through. Bleed-through can result when Europium and Terbium Acceptor-beads emit signal at the same time but the filter used to separate out the signal of one specific Acceptor-bead also lets light pass from the second Acceptor-bead (see Additional file 3: Figure S3 for graphical representation of the principle). We measured Terbium and Europium signal of two different conditions where one condition had no analyte and no second Acceptor-bead (background noise) while in the second condition analyte was added. The signals resulting from those measurements were used to calculate the percent of bleed-through. The bleed-through for the Terbium filter was estimated at 2.4 % (Fig. 4e), while the bleed-through of the Europium filter was estimated to be 0.9 % (Fig. 4f). All measurements reported for the steric hindrance in this section as well as all other data referring to the duplex assay is corrected for filter bleed-through based on these estimates.

**Fig. 5:**
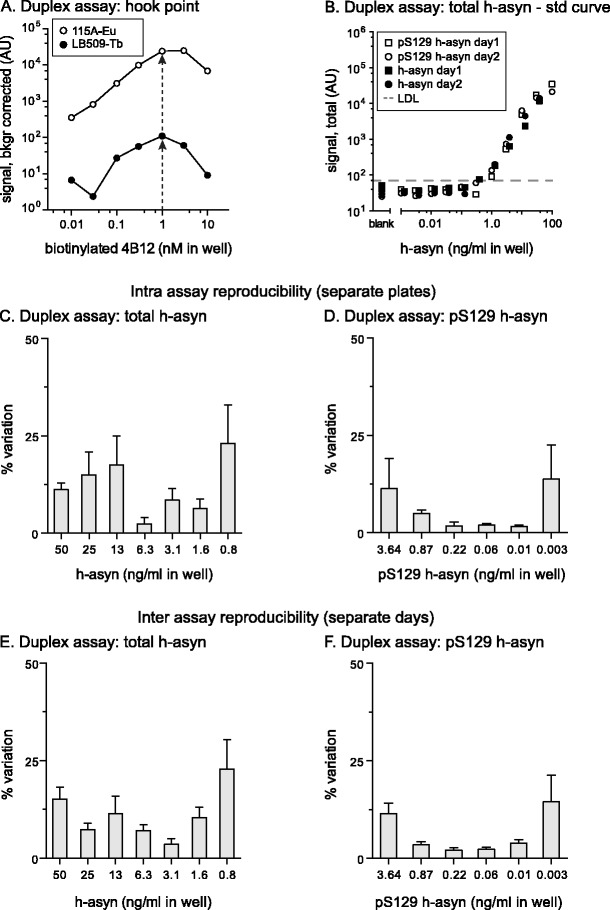
Characterization of total and phosphorylated S129 human alpha-synuclein duplex assay*.* The hook-point for biotinylated 4B12 antibody was determined simultaneously in a mix of Europium Acceptor-bead coupled 11A5 antibody and Terbium Acceptor-bead coupled LB509 antibody (**a**). Arrows indicate optimal 4B12 concentrations for both Acceptor-bead variants. Equivalency of the signal obtained in the total h-asyn assay was demonstrated for pS129 h-asyn and h-asyn proteins in the duplex assay in two consecutive experiments and data plotted in panel (**b**). All protein standards were spiked in naïve rat brain lysate. Lower detection limit (LDL) was 450 pg/ml and is indicated with a dashed line. Intra assay variation was calculated based on three standard curves which were measured on the same day but on separate plates for each emission signal separately (**c** and **d**). Inter assay variation data was calculated based on six pairs of standard curves performed on separate days (**e** and **f**). *AU* arbitrary units

Hook-point was established for the duplex assay where Europium and Terbium signal was measured in the same well. Optimal biotinylated 4B12 concentration was found to be unchanged at 1.0 nM (Fig. 5a). To ensure that the Donor-beads added to the wells were sufficient to provide enough oxygen singlets for two Acceptor-beads, a serial dilution of Donor-beads was made ranging from 6 to 40 μg/ml in the well. No change in signal intensity for either Acceptor-bead was seen (Additional file 4: Figure S4). When comparing the signal intensity of h-asyn and pS129 h-asyn standard curves both measured using the total h-asyn assay (Terbium channel), no difference in signal intensity or curve shape was observed (Fig. 5b). While the LDL of the pS129 h-asyn duplex assay was similar to the LDL obtained for the pS129 h-asyn uniplex assay, we observed a 235-fold increase (from 2 to 470 pg/ml) when comparing the LDL for uniplex and duplex total h-asyn assays.

Intra- and inter-assay variations were calculated between six individual standard curves performed on three different days using 6 to 7 concentrations on those curves to validate the accuracy and variation between runs. For the total h-asyn assay, average intra assay variation did not exceed 23 % while the average inter assay variation was lower than 22.8 % (Fig. 5c, e). Values were notably higher than what was obtained with the uniplex total h-asyn assay. For the pS129 h-asyn assay, average intra-assay variation did not exceed 13.7 % while the average inter assay variation was lower than 14.5 % (Fig. 5d, f). Z’ values were calculated using 12 replicates of naïve rat brain lysate (blank) or lysate spiked with pS129 h-asyn protein. Z’ values for the total h-asyn and the pS129 h-asyn assay was 0.81 and 0.84, respectively.

### Quantification of pS129 and total human alpha-synuclein in HEK293 cells co-expressing h-asyn and polo-like kinase 2

**Fig. 6:**
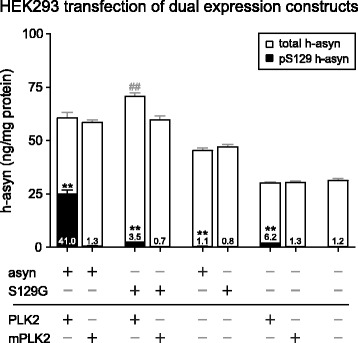
S129 specific phosphorylation of human alpha-synuclein using PLK2 in vitro*.* HEK293 cells were transfected with dual expression plasmids coding for h-asyn or S129A h-asyn as well as PLK2 or the kinase dead mutant (mPLK2) (*n* = 6 per group; about 3 μg of protein loaded). Levels of total (open) and pS129 h-asyn (filled) of whole cell lysates were measured using the newly established AlphaLISA duplex assay and are presented in overlapping bars. Percentages of pS129 h-asyn to total h-asyn are indicated in the bottom of respective bars. **p <0.01; ##*p* <0.01; two-tailed Mann-Whitney test; *Error bars* indicate SEM

To further validate the newly established duplexing assay we co-transfected HEK293 cells with a single vector construct co-expressing h-asyn and PLK2 under the human CMV and murine CMV promoters, respectively (*n* = 6 per group). PLKs have been shown previously to highly phosphorylate asyn specifically at position 129 [26]. As controls, a PLK2 kinase dead-mutant (mPLK2) and non-phosphorylatable S129G h-asyn were used. All proteins were also expressed in combination with YFP or mCherry instead of h-asyn or PLK2, respectively. Note, HEK293 cells produce a substantial amount of endogenous h-asyn as well as pS129 h-asyn (1.2 % of total h-asyn), which can be measured using the duplexing assay (Fig. 6). Expression of PLK2 without h-asyn showed an increase in pS129 h-asyn by 4.8-fold due to PLK2 activity compared to single mPLK2 expression but did not phosphorylate h-asyn efficiently/completely. Single h-asyn also resulted in a smaller yet significant increase of pS129 h-asyn compared to expression of S129G h-asyn alone since more phosphorylatable h-asyn is available for endogenous kinases. Expressing S129G h-asyn in combination with PLK2 resulted in an increase in pS129 h-asyn by 5.2-fold due to PLK2 activity on endogenous h-asyn but was considerably smaller (11.7-fold) than in the h-asyn + PLK2 group. When h-asyn is co-expressed with PLK2, pS129 h-asyn levels increased as expected when compared to h-asyn expressed together with mPLK2 (32.4-fold). No difference in total h-asyn levels was observed.

### Evaluation of human alpha-synuclein phosphorylation efficiency in the rat brain by viral vector co-expression of h-asyn and polo-like kinase 2

**Fig. 7:**
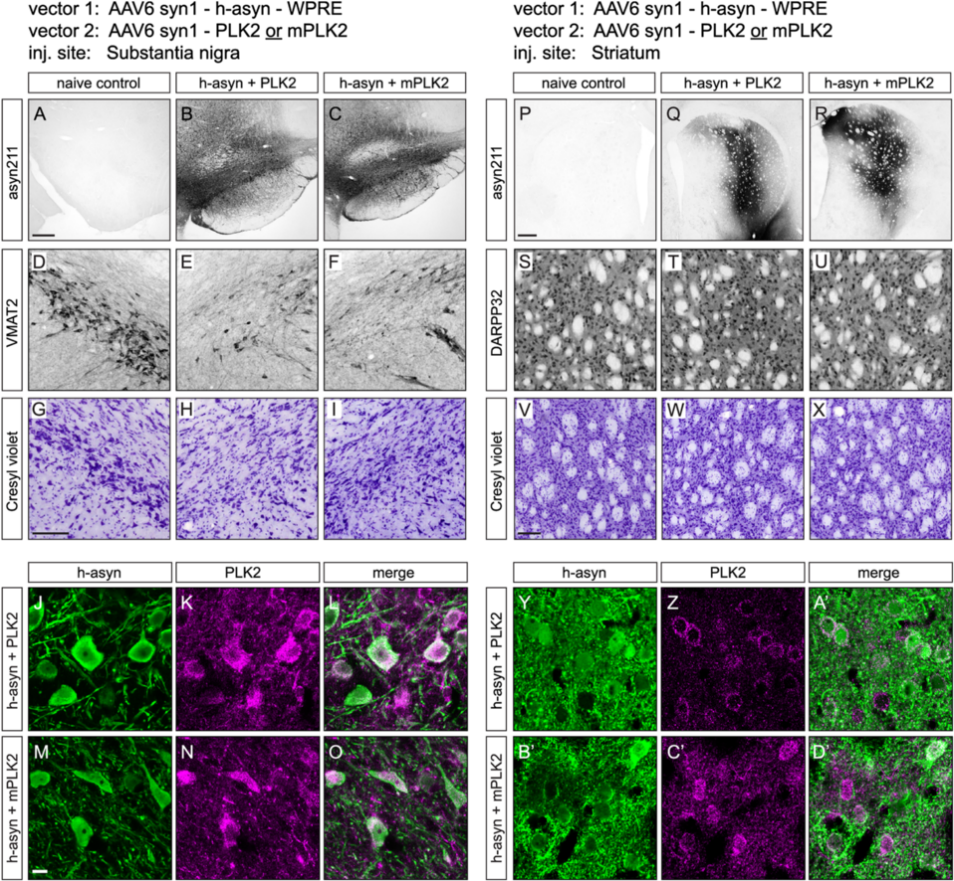
Transgene expression after rAAV injection in the rat substantia nigra and striatum. Adeno-associated viral vectors expressing either PLK2 or mPLK2 were mixed at a 1:1 ratio with a vector encoding for h-asyn and injected into rat substantia nigra or striatum (*n* = 4 per group). Two weeks after transduction, gene expression was visualized using the asyn211 antibody on sections from ventral midbrain (**a**–**c**) or striatum (**p**–**r**). Panels (**d**–**f**) and (**s**–**u**) show vesicular monoamine transporter 2 (VMAT2) stainings for dopaminergic nigral neurons and dopamine- and cAMP-regulated neuronal phosphoprotein 32 (DARPP32) stainings for striatal neurons from the two targeted structures, respectively. *Cresyl violet* stainings from adjacent sections are shown in (**g**–**i**) for the ventral midbrain and in (**v**–**x**) for the striatum. High magnification confocal images of transduced cells illustrate co-expression of asyn (*green*) and PLK2 (*magenta*) transgenes in nigral (**i**–**o**) and striatal neurons (**y**–**d**’). Scale bar in (**a**) and (**p**) is 0.4 mm and applies to (**a**–**c**) and (**p**–**r**), in (**g**) and (**v**) are 100 μm and apply to (**d**–**i**) and (**s**–**x**), respectively, and in (**m**) is 10 μm and applies to (**i**–**o**) and (**y**–**d**)'

To investigate the effects and efficiency of PLK2 induced pS129 of h-asyn in vivo we used a 1:1 mix of recombinant adeno-associated viral vector serotype 6 (rAAV6) expressing h-asyn together with rAAV6 expressing either PLK2 or mPLK2. Vector mixes were injected into the SN (Fig. 7a–o) or striatum (Fig. 7p–d’) of rats to examine h-asyn handling and phosphorylation differences in two different cell types. Targeting and expression were assessed two weeks after injection (*n* = 4 per group) by staining coronal ventral midbrain and striatal sections using the h-asyn specific antibody syn211 (Fig. 7a–c, p–r). H-asyn staining showed a good coverage and transduction of ventral midbrain neurons (Fig. 7b, c) while striatum was covered partially (Fig. 7q, r). Presence or absence of cell death was visually assessed first using two cell type specific stainings for dopaminergic neurons (Fig. 7d–f) and striatal neurons (Fig. 7s–u), and cresyl violet staining of adjacent sections (Fig. 7g–i, v–x). Initial visual inspection suggested a partial reduction of dopaminergic neurons on the injected compared to uninjected side in the SN (Fig. 7d–i). As we have chosen a low vector titer and a short expression time, we did not anticipate any major cell loss and found no significant difference between the PLK2 and mPLK2 group (*p* = 0.11). In striatal sections, no apparent cell death was found in either experimental group when compared to a naïve brain (Fig. 7s–x) nor was there any significant change found between the PLK2 and mPLK2 group (*p* = 0.49). To confirm double transduction of single neurons, sections were co-stained for h-asyn and PLK2 (Fig. 7j–o, y–d’). Staining for h-asyn showed similar localization of the protein in nigral (Fig. 7j, m) and striatal (Fig. 7y, b’) neurons. PLK2 (Fig. 7k, z) or mPLK2 (Fig. 7n, c’) staining co-localized in both brain areas with that of h-asyn (Fig. 7l, o, a’, d’) showing that both PLK2 proteins and h-asyn are co-expressed in the same targeted cells.

**Fig. 8:**
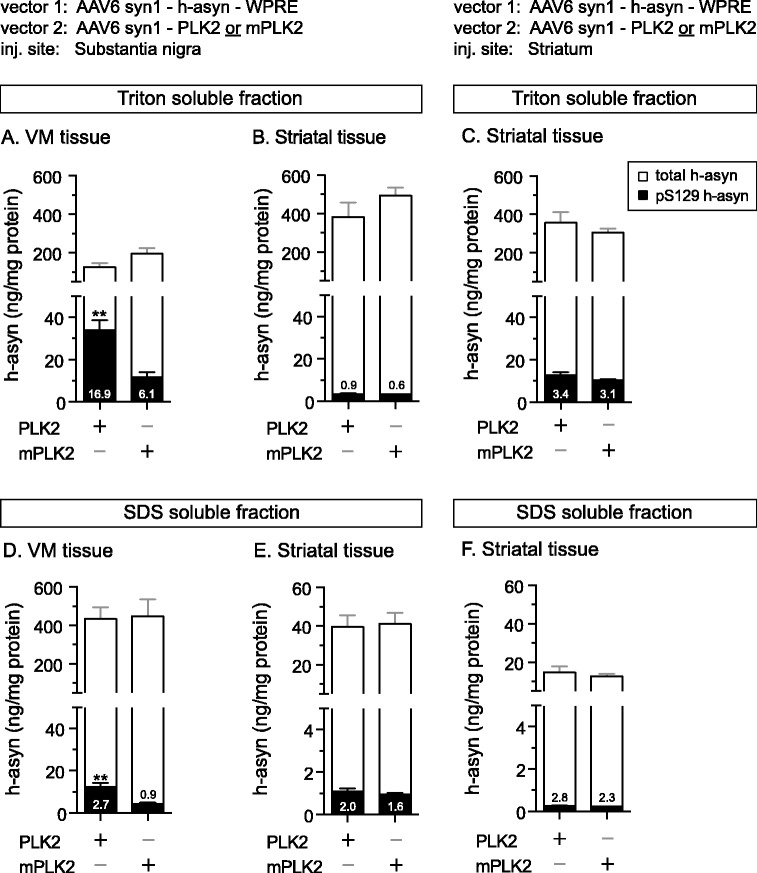
Assessment of S129 phosphorylated human alpha-synuclein in rat brain tissue. Quantification of pS129 h-asyn species after overexpression from rAAV6 vectors in the substantia nigra (**a**, **b**, **d**, **e**) or striatum (**c**, **f**). Tissue pieces (*n* = 6 per group) were processed sequentially with 1 % Triton (about 300 ng for striatal and 90 ng for VM samples of protein loaded) (**a**–**c**) followed by 1 % SDS (about 600 ng of protein loaded) (**d**–**f**) containing lysis buffer. Total (open) and pS129 (filled) h-asyn levels are presented in overlapping bars. Numbers inside the bars show the percent of pS129 to total h-asyn. **p <0.01; two-tailed Mann-Whitney test; *Error bars* indicate SEM

To quantify total and pS129 h-asyn levels of transduced rat brain areas, fresh frozen tissue (*n* = 6 per group) taken two weeks after injection was lysed using first 1 % Triton followed by 1 % SDS lysis buffer sequentially and samples of each fraction and region were analyzed using the newly established duplex assay (Fig. 8). No differences in total h-asyn levels were observed in ventral midbrain samples for both Triton and SDS fractions (Fig. 8a, d) nor in fractions of striatal tissue containing the terminals of transduced nigral neurons (Fig. 8b, e). An increase of pS129 h-asyn levels of 2.7-fold was found in ventral midbrain neurons expressing PLK2 when compared to cells expressing mPLK2 (Fig. 8a). This increase was not only visible in the Triton but also in the SDS fraction (Fig. 8d). No increase of pS129 h-asyn levels was found in terminals of nigral neurons (Fig. 8b, e). The levels of h-asyn in striatal neurons were unchanged between groups and fractions (Fig. 8c, f). Interestingly, striatal neurons expressing both h-asyn and PLK2 did not show an increase of pS129 h-asyn levels suggesting a different regulatory mechanism of h-asyn phosphorylation or PLK2 activity between striatal and ventral midbrain neurons.

### Alterations in phosphorylation state of human alpha-synuclein (h-asyn) in Parkinson’s disease (PD) patients with and without dementia

**Fig. 9:**
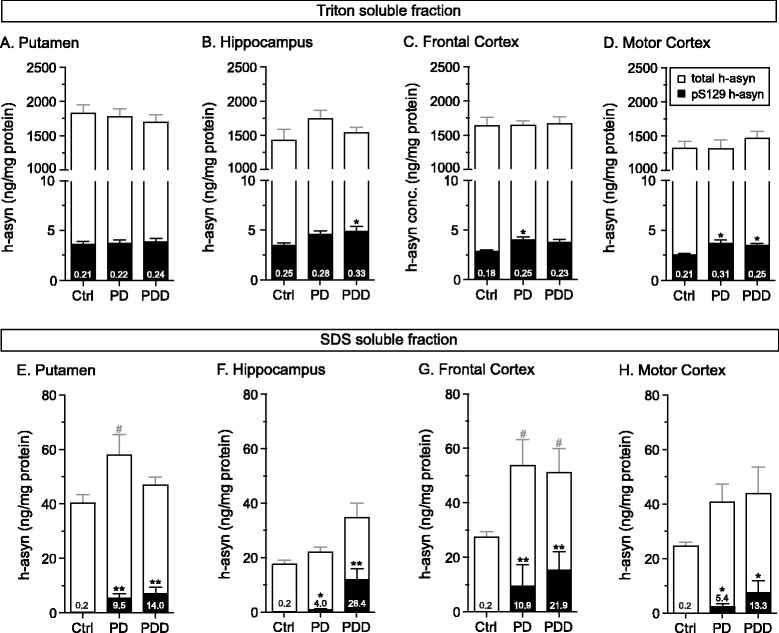
Assessment of S129 phosphorylated human alpha-synuclein species in post mortem brain tissue. Frozen human brain tissue samples were received from healthy controls (Ctrl), patients diagnosed with Parkinson’s disease (PD) or Parkinson’s disease with dementia (PDD) (*n* = 8 per group). Four brain regions were analyzed: putamen (**a**, **e**), hippocampus (**b**, **f**), frontal cortex (**c**, **g**) and motor cortex (**d**, **h**). Tissue was sequentially lysed first using 1 % Triton (about 100 ng of protein loaded) (**a**–**d**) followed by 1 % SDS containing lysis buffer (about 2 μg of protein loaded) (**e**–**h**). Total (open) and pS129 (filled) h-asyn levels are presented in overlapping bars. Numbers inside the bars show the percent of pS129 to total h-asyn. *p <0.05; **p <0.01; #*p* <0.05; one-way ANOVA followed by a Tukey’s HSD test or Kruskal-Wallis followed by a Dunn’s multiple comparisons test; *Error bars* indicate SEM

First, we set out to evaluate the sample reproducibility for the AlphaLISA duplexing assay. To do so, we lysed frontal cortex tissue samples from healthy controls (*n* = 16) using buffer containing 1 % Triton and measured total as well as pS129 h-asyn as five independent measurements in each sample carried out over several days. We found that the variation in the total and pS129 h-asyn values were very similar despite 2–3 orders of magnitude differences in their quantities and were below 25 % in all but 1 measurement (Additional file 5: Figure S5). Furthermore, when considering the ratio between the pS129 and total h-asyn, we found that the variation of these estimates were even lower (76 out 80 estimates with <10 %) (Additional file 5: Figure S5). We then continued to investigate the accumulation of pS129 h-asyn in human brain tissue samples from patients diagnosed with either PD alone or PD with dementia (PDD) as well as in samples obtained from healthy controls (Ctrl) (*n* = 8 per group). We acquired four different brain areas for each case – putamen, hippocampus, frontal cortex and motor cortex – which were sequentially lysed first with buffer containing 1 % Triton followed by 1 % SDS containing buffer. Both fractions were analyzed using the newly validated duplex AlphaLISA assay (Fig. 9). When looking at the triton fraction, no difference was observed for total h-asyn but pS129 h-asyn levels were significantly increased for PD patients in both cortices and for PDD patients in hippocampus and motor cortex when compared to Ctrl cases (Fig. 9a–d). Evaluation of SDS soluble h-asyn showed a significant accumulation of total h-asyn levels in the putamen and frontal cortex for PD cases as well as in the frontal cortex for PDD cases when compared to Ctrl samples (Fig. 9e, g). Additionally, in all four brain areas a significant increase of pS129 h-asyn quantities was detected for both PD and PDD cases compared to Ctrl cases (Fig. 9e–h). Notably, pS129 h-asyn levels in hippocampal samples were substantially increased in PDD cases when compared to PD cases (Fig. 9f) signifying the importance of the hippocampal pathology in demented PD patients.

## Discussion

Several asyn specific assays have been developed to measure levels of total asyn protein and its modified variants in brain tissue, CSF and plasma samples with the goal to find a wet biomarker for PD and other synucleinopathies [17, 18, 21, 27, 28]. As pS129 asyn comprises a large part of asyn found in inclusions [14], it has been of interest to further measure pS129 asyn levels in various samples collected from patients. However, so far only four studies have quantified the levels of pS129 asyn in brain tissue [15, 25, 29, 30] while five studies investigated CSF and blood samples [22–24, 31–33]. Notably, none are duplexed to report total and pS129 asyn simultaneously in the same sample.

By using the newly developed Terbium Acceptor-beads (PerkinElmer, USA), we developed a specific and sensitive duplex AlphaLISA assay to quantify total and pS129 h-asyn in the same well (Additional file 2: Figure S2), thereby providing a highly accurate phosphorylation ratio. This is especially important since pS129 asyn levels are under normal conditions lower than 0.4 %. Our duplex assay proved useful not only in cell culture lysates but also in human and humanized rat brain tissue samples. Even when as little as 0.2 % of total h-asyn was phosphorylated at S129, the dynamic range of the duplexing assay was sufficiently wide to obtain both measurements with a single dilution and run. The assay protocol requires no washing and can be completed within 90 min of incubation time, which is therefore ideal for high throughput screenings, allows for cost efficient runs and uses lower amounts of sample material than running separate uniplex assays.

Each (uniplex) component of the assay developed here has a matching or improved sensitivity compared to previously described results. Naturally, in combining two uniplex assays into one duplex assay, we expected a decrease in sensitivity of one of the assays due to the use of the Terbium as opposed to the Europium Acceptor-beads. In order to minimize and eliminate any significance of the S/B disadvantages due to the difference in signal intensities emitted by the two beads, we implemented the weaker of the two signals (Terbium beads) for the more abundant of the two species, namely the total h-asyn protein. Nevertheless, in future studies, this S/B disadvantage could be improved by, e.g., implementation of an enhanced filter/detector system to better channel and amplify the low signal generated by the Terbium Acceptor-beads and/or by a development of a version of the Terbium Acceptor-beads with superior signal characteristics. This would not only result in lower standard deviation values between replicates and therefore more accurate estimations but also a lower detection limit and wider linear response range in the assay.

As already noted by others [20, 34], the quality (and therefore the estimated quantity) of recombinant asyn proteins used as standards in an assay is important especially as different production batches are used between laboratories and sometimes also within one laboratory. Bidinosti and colleagues reported up to 3-fold differences in assay signal depending on the protein standards used [20]. Standards generated by phosphorylating recombinant h-asyn protein in vitro using PLK2, as is done here, have been reported to result in incomplete phosphorylation of the protein at the S129 residue [26], potentially with batch to batch variations, and present the risk for incorrectly calculated phosphorylation ratios. Therefore, a quality control step such as isoelectric focusing gel or alternatively mass spectrometrical analysis should be applied in order to evaluate phosphorylation efficiency of each recombinant protein batch. A standardized and careful estimation of protein concentration and phosphorylation would allow more reproducible results between batches used in a given assay platform and various types of assays targeting the same protein.

Previous studies have suggested that PLK2 not only phosphorylates asyn [26, 30, 35] but also targets it for autophagosomal degradation [36]. Here, we could readily demonstrate that PLK2 can phosphorylate h-asyn in co-transfected HEK293 cells. Although, we found no evidence for reduction in total h-asyn levels in this cell-based in vitro assay nor was this apparent in rat brain tissue co-expressing h-asyn and PLK2 proteins. Nevertheless, we found an elevation of pS129 h-asyn levels from 6.1 to 16.9 % (2.8-fold) in rat ventral midbrain neurons caused by the co-expression of PLK2. Furthermore, we determined the baseline pS129 rat asyn (r-asyn) level in the Triton fraction of the ventral midbrain samples to be 0.007 % (Additional file 6: Figure S6) suggesting that the transgenic h-asyn is phosphorylated about 890 times more than the endogenously expressed r-asyn. Notably, our estimations of baseline pS129 to total r-asyn ratio is lower than what Bergeron and colleagues reported [30]. Nevertheless, the large difference between the proportions of endogenous pS129 r-asyn versus transgenic pS129 h-asyn protein in vivo suggests an alternative handling of the h-asyn protein. This difference is unlikely to be due to excessive overexpression of asyn per se as the transgenic human protein concentration in the tissue was 233 ng/mg protein, while the endogenous rat protein levels were estimated at an average of 706 ng/mg protein (Additional file 6: Figure S6).

In the striatal tissue, the endogenous r-asyn levels (1843 ng/mg protein) are about 2.6-fold higher than in the ventral midbrain with about 0.013 % being phosphorylated at S129. AAV vector mediated expression of h-asyn in the striatum resulted in an additional 336 ng/mg of asyn protein and its phosphorylated fraction was found to be 3.1 %. Taken together, these observations suggest that the two cell populations in striatum and midbrain respond to the transgenically expressed h-asyn in a similar way, namely the hyper-phosphorylation of h-asyn. Interestingly, the co-expression of PLK2 in the striatal neurons did not result in an increase in the pS129 h-asyn portion, nor were there any indications that the total h-asyn levels in this group was reduced, making it unlikely for differential degradation of the pS129 species in the striatal neurons to account for this difference. Taking into consideration that most of the asyn found in LBs is phosphorylated at the S129 residue and that this post-translational modification is suggested to promote the formation of oligomeric species in vitro [14, 15], our findings could provide evidence as to why certain cell types such as dopaminergic neurons are more vulnerable and affected earlier by asyn toxicity than cell types located in the striatum or cortical areas [37].

Another striking observation here was that the proportion of phosphorylated h-asyn in the striatal axon terminals of the nigrostriatal neurons was lower than in the cell bodies located in the midbrain (0.6 % versus 6.1 %). Although the co-expression of PLK2 in nigral neurons resulted in readily detectable increases in the portion of pS129 h-asyn, there was no measurable effect at the level of their terminals. This was true despite the fact that transportation of the transgenic protein from the cell bodies to the terminals was rather efficient. These observations could suggest either a selective transport of the non-phosphorylated h-asyn to the terminals, or alternatively an effective de-phosphorylation once the protein leaves the cell body; entering the transport system. The former scenario would argue that the two pools of asyn proteins have differential functions in the cells (e.g., non-phosphorylated asyn is selectively involved in vesicle pool regulation and SNARE complex formation [38, 39]); alternatively the second scenario could point to differential phosphatase activities in the ventral midbrain and the striatum, although the precise composition and relative activity of phosphatases that act on pS129 asyn are not well studied.

Although the total amount of transgenically expressed h-asyn was about 33 % of endogenous asyn levels in the Triton fraction of the midbrain tissue, we found nearly 70-fold increase in the SDS fraction in the same samples (for endogenous r-asyn measurements see Additional file 6: Figure S6). Notably this striking difference was detected not only in the total asyn protein but also in the pS129 species, although the great majority of all asyn in the SDS fraction was not phosphorylated. On the other hand, the contribution of h-asyn in the striatal axon terminals in the Triton insoluble fraction was comparable to the endogenous r-asyn. Since animals in this experiment were killed 2 weeks after injection, these findings could suggest evidence of early formation of h-asyn species resistant to Triton treatment that is independent of phosphorylation at serine 129 residue. An alternative explanation that is not mutually exclusive with the above scenario is that h-asyn is translocated to the nucleus and thus appears in the SDS extracted fraction. In fact, when samples are lysed sequentially first in Triton and then in SDS containing buffers, as done here, proteins located in the nucleus such as Histone3 are released to the supernatant in the SDS fraction since Triton cannot break the nuclear membrane. While this increase was evident in ventral midbrain neurons, a similar increase in Triton insoluble h-asyn was not observed in striatal neurons – i.e., the levels of h-asyn in the SDS fraction were comparable to those of endogenous r-asyn. Notably, independent of the source (compartment) of the SDS extracted asyn protein, it appears from our observations that even a robust increase in the pS129 post translationally modified species has no effect on the relative abundance of this protein in the Triton soluble and insoluble asyn pool in the tissue. In other words, addition of the active PLK2 enzyme increases the pS129-to-total asyn ratio by about 3-fold in both fractions.

Total amounts of Triton soluble asyn in naïve rats was comparable to levels in post-mortem human brain tissue, while the SDS fraction showed higher amounts of total asyn in humans compared to rats. Although the same relationship was true for the fraction of asyn that is phosphorylated in the rat and human brain, respectively, when the h-asyn is expressed in the rat brain, this protein species was phosphorylated to a higher level than either of the endogenous species. In human post-mortem brain tissue, we found no change in total or h-asyn levels in the Triton fraction between healthy control and disease cases and in 4 brain areas studied here. Similarly, pS129 asyn ratio was between 0.2 and 0.3 % of the total asyn, where only small differences appeared in PD and PDD cases against the controls, with no clear distinction between two disease states. The findings in the SDS soluble fraction were fundamentally different: First, the pS129 species was over-represented in this fraction by 20 to 100-fold in the PD/PDD cases. This increase is in agreement with previous studies [40, 41] and reflects the pathological features of a diseased brain. Secondly, a clear difference between two disease groups could be seen in the hippocampal area, where the pS129 species were much more abundant in the PDD brains. Notably, analysis in the putaminal tissue did not reveal a difference and in other cortical areas (motor and frontal cortices) the differences were smaller and did not reach significance in this dataset. This result corroborates our previous finding where a specific increase in the number of h-asyn positive Lewy neurites was found in hippocampal region on histological specimens of PDD cases when compared to PD cases [42].

## Conclusion

In summary, we have established a novel assay which simultaneously measures total and pS129 h-asyn levels and therefore increases the accuracy of the quantified phosphorylation ratio. Using this assay, we examined the extent of PLK2 enzyme activity on asyn in vitro and in vivo and showed that PLK2 increases pS129 h-asyn levels in HEK293 cells. Notably, when rat brain cells were transduced to co-express h-asyn and PLK2, pS129 h-asyn quantities increased in the ventral midbrain but not in the striatum. Furthermore, we measured the asyn phosphorylation state in post-mortem brain areas of PD and PDD cases allowing us to see a clear increase in pS129 h-asyn signal in the hippocampus when PD patients develop dementia. Future studies will need to focus on the simultaneous measurement of total and pS129 h-asyn levels in multiple biospecimens from the same patient (CSF and/or plasma as well as brain tissue) and to correlate those results to brain asyn levels and disease progression and severity.

## Methods

### The AlphaLISA® platform

The assay was developed by PerkinElmer as a no-wash assay for a highly sensitive, high throughput, widely dynamic and robust detection of analytes in biological samples [43]. It offers several advantages over conventional enzyme-linked immunosorbent assays (ELISAs). The signal of this bead-based immunoassay is based on the luminescent proximity principle using oxygen-channeling chemistry. In the most commonly used format, the Donor-bead is coupled *via* streptavidin-biotin link to the first antibody while the second antibody is directly coupled to the Acceptor-bead (Additional file 7: Figure S7). Binding of both antibodies to the same analyte brings the two beads into desired proximity. Excitation of Donor-beads at 680 nm creates singlet oxygens triggering a cascade of chemical events in Acceptor-beads less than 200 nm away leading to a chemiluminescent emission. The assay is performed with a simple “mix-and measure” protocol shown in Additional file 7: Figure S7.

### Establishment of the human alpha-synuclein specific assays

Antibodies were obtained from commercial suppliers or as a gift (as indicated in Table 1) either as manufactured or custom orders to ensure antibodies were at 1 mg/ml in only PBS (Thermo Fisher Scientific, USA) with no BSA or azide present in the solution.

Biotinylation of antibodies: 2 mg/ml NHS-ChromaLink-biotin (SoluLink, USA) in PBS pH 7.4 was added to each antibody solution (BSA, glycerol and azide free) at a 30:1 molar ratio. The volume was adjusted to 100 μl with PBS pH 7.4. After incubating for 2 h at 23 °C in the dark, the antibody-biotin solutions were filtered through Zeba spin columns (Thermo Scientific, USA) at 1,500 rcf for 2 min to remove unbound biotin. Concentration of the antibody and biotinylation efficiency were measured using a NanoDrop2000 instrument (Thermo Fisher Scientific, USA). Optical densities (OD) values at 280 and 354 nm were used to assess total protein and biotin concentrations, respectively. Note that the biotinylation efficiency, estimated here indirectly *via* ChromaLink measurements, varies between antibodies due to the abundance of Lysine residues. For the conjugations performed in this experiment, we found an up to 3-fold difference between individual antibodies. The presence of aggregated antibody was assessed at 450 nm. All values obtained in our experiments were below 0.01 (The supplier recommends OD value <0.1 as acceptable). Biotinylated antibodies were stored at 4 °C at a concentration of 500 nM in PBS with 0.05 % NaN_3_ and 0.1 % Tween-20.

Acceptor-bead coupling of antibodies: 0.3 mg (for antibody screening) or 2 mg (for frequently used batches) of Europium Acceptor-beads (AlphaLISA® Acceptor-beads, PerkinElmer, USA) or Terbium Acceptor-beads (AlphaPlex™ 545 Acceptor-beads, PerkinElmer, USA) were washed with PBS, centrifuged at 16,000 rcf for 15 min and re-suspended in a 10:1 bead to antibody weight ratio. The volume was adjusted to 60 μl or 400 μl, respectively, with 0.13 M phosphate buffer pH 8.0. 10 % Tween-20 was added to reach a final concentration of 0.06 % and freshly prepared 25 mg/ml NaBH_3_CN in H_2_O to reach a final concentration of 1.18 mg/ml. Afterwards the antibody-bead solution was incubated for 48 h at 37 °C. To block unreacted sites freshly made 65 mg/mL CMO in a 0.8 M NaOH was added at a final concentration of 3 mg/ml and incubated for 1 h at 37 °C. The antibody-bead solution was centrifuged for 15 min at 16,000 rcf and washed twice with 60 μl or 400 μl, respectively, of 0.1 M Tris-HCl pH 8.0. Antibody-coupled Acceptor-beads were re-suspended at 5 mg/mL in storage buffer (PBS + 0.05 % Proclin-300), vortexed, briefly sonicated (5 min in Branson1210 sonication water bath) and stored at 4 °C.

In all steps below, antibodies and beads were diluted in 1× assay buffer (10× AlphaLISA Immunoassay buffer: 250 mM HEPES, pH 7.4, 1 % Casein, 10 mg/mL Dextran-500, 5 % Triton X-100 and 0.5 % Proclin-300, PerkinElmer, USA) and samples were diluted using PBS pH 7.4.

To screen antibody pairs for the h-asyn specific assay, a fixed concentration of 100 μg/ml h-asyn overexpressing rat brain lysate was used as a positive control. As negative control (background) 100 μg/ml wild-type rat brain lysate was used. For the pS129 h-asyn specific assay, a final concentration of 14 ng/ml pS129 h-asyn or 50 ng/ml S129A h-asyn protein standard was added to 10 μg/ml asyn knockout mouse brain lysate (matrix) and used as positive and negative analyte, respectively. Each biotinylated antibody was combined with each Europium Acceptor-bead coupled antibody.

### Uniplex AlphaLISA optimization

Hook-point of biotinylated antibodies was determined by serially diluting the biotinylated antibody 1:3 with a 50 μg/ml Europium Acceptor-bead solution resulting in seven in-well dilutions ranging from 10 to 0.01 nM. To 10 μg/ml asyn knockout mouse brain lysate, 50 ng/ml of h-asyn protein as a positive analyte or mouse asyn protein as a negative analyte (background) was added. For the pS129 h-asyn specific assay, 14 ng/ml of pS129 h-asyn protein was used as positive analyte or S129A h-asyn protein as negative analyte (background) and spiked into 10 μg/ml asyn knockout mouse brain lysate. Donor-beads (AlphaScreen® streptavidin-coated Donor-beads, PerkinElmer, USA) were added at a final concentration of 40 μg/ml.

Assay run protocol: 5 μl of sample were added into a 384-well AlphaPlate (PerkinElmer, USA). Each sample was run in triplicate (except blanks which were run in nine replicates) and a relative standard deviation between sample triplicates of <11 % and between blank replicates of <21 % was accepted. A concentration of 50 μg/ml Europium Acceptor-beads coupled to antibodies (1:100 dilution from stock) and 5 nM biotinylated antibody (1:100 dilution from stock; except for hook-point analysis) were mixed together beforehand and 5 μl were added to each well. After 1 h of incubation in the dark, 15 μl of Donor-beads (AlphaScreen® streptavidin-coated Donor-beads, PerkinElmer, USA) at a concentration of 66.7 μg/ml (1:75 dilution from stock; except for Donor-bead optimization) were added to each well. All pipetting steps were done using the Janus pipetting robot (PerkinElmer, USA). After an incubation of 30 min in the dark, the plate was read using the EnVision 2104 Multilabel plate reader (PerkinElmer, USA). The standard AlphaScreen emission filter (CW 570 nm) was used for single emission assays.

Standard curves were established by serially diluting the stock of recombinant proteins (1.9 μg/ml for h-asyn protein, 1.4 μg/ml for pS129 h-asyn and 5 μg/ml for remaining recombinant proteins) 1:3 using 10 μg/ml rodent brain lysate resulting in 11 dilutions ranging from 28 ng/ml to 0.28 pg/ml for pS129 h-asyn and 39.2 ng/ml to 0.39 pg/ml for h-asyn in-well synuclein concentration. Un-spiked lysates were used as blanks and were run in nine replicates.

### Duplex AlphaLISA optimization

Both Europium Acceptor-beads (AlphaLISA® Acceptor-beads, PerkinElmer, USA) or Terbium beads (AlphaPlex™ 545 Acceptor-beads, PerkinElmer, USA) were used to develop a duplexing assay. Each signal measurement was corrected for filter bleed-through. Hook-point was established as described above.

Assay run protocol was modified so 50 μg/ml Europium Acceptor-beads coupled to antibodies as well as 50 μg/ml Terbium Acceptor-beads coupled to antibodies (for both 1:100 dilution from stock) and 5nM biotinylated antibody (1:100 dilution from stock; except for hook-point analysis) were mixed together beforehand and 5 μl were added to each well. All pipetting steps were done using the Janus pipetting robot (PerkinElmer, USA). A relative standard deviation between sample triplicates for Europium channel of <13 % and for Terbium channel of <27 % was accepted. Relative standard deviation between blank replicates for Europium channel <19 % and for Terbium channel of ≤40 % was accepted. The plates were read using first the Resorufine/Amplex Red FP535 filter for Terbium signal and then again using the Europium 615 filters for the Europium emission.

Standard curves were acquired by serially diluting the stock of recombinant proteins (1.9 μg/ml for h-asyn protein, 1.4 μg/ml for pS129 h-asyn and 5 μg/ml for remaining recombinant proteins) 1:2 using either 10 μg/ml rodent brain lysate or appropriate dilutions of un-treated HEK293 cell lysates resulting in 11 or 18 dilutions ranging from 100 ng/ml to either 0.21 pg/ml in-well asyn concentration. Un-spiked lysates were used as blanks and were run in nine replicates.

Donor-bead optimization for duplexing assay was achieved by serially diluting the Donor-beads with 1× assay buffer to 4 different concentrations (200, 130, 70 and 30 μg/ml). As positive analyte 14 ng/ml of pS129 h-asyn protein or as negative analyte mouse asyn protein (background) was spiked into 10 μg/ml asyn knockout mouse brain lysate.

Steric hindrance and filter bleed-through for duplexing assay was tested by adding either PBS or 11A5 coupled Europium Acceptor-beads (50 μg/ml final concentration) to a stock solution containing 5 nM biotinylated 4B12 and 50 μg/ml LB509 coupled Terbium Acceptor-bead and vice versa. As analyte 14 ng/ml of pS129 h-asyn protein was added to 10 μg/ml asyn knockout mouse brain lysate. Un-spiked lysate was used as blank.

### Recombinant alpha-synuclein protein standards

Glutathione S-transferase (GST)-tagged recombinant h-asyn was obtained from MyBioSource, USA (MBS957854). The remaining recombinant protein standards – pS129 h-asyn, S129A h-asyn and m-asyn were produced as described below.

#### For pS129 h-asyn

Recombinant protein was produced as described elsewhere [22, 44, 45]. Briefly, the pGEX-4T1 vector containing a GST h-asyn fusion gene was inserted into *E.coli* BL-21 bacteria for protein expression. The transformed bacteria were grown in Luria Bertani (LB) medium. Glutathione sepharose 4B beads were used to capture the GST h-asyn proteins in the bacterial lysate and the GST-tag was cleaved by thrombin. The molecular weight and purity of the h-asyn proteins were confirmed by SDS-PAGE gel and the concentration estimated using a BCA protein assay (Pierce Biotechnology, Rockford, IL).

Purified h-asyn was phosphorylated at S129 in vitro by adding 1 μg PLK2 protein to 1.44 mg/ml (100 μM) h-asyn in kinase reaction buffer (20 mM HEPES, 1.09 mM ATP, 2 mM DTT, 10 mM MgCl_2_, pH 7.4). Reaction mixture was incubated at 30 °C for 24 h. Phosphorylation efficiency of h-asyn was determined by loading 300 ng protein on isoelectric focusing gels pH5-8 (Bio-Rad, USA) to separate pS129 h-asyn from non-phosphorylated h-asyn by charge. Gels were ran for 30 min at 100 V, 30 min at 250 V and 1.5 h at 500 V and afterwards incubated in Western Blot (WB) running buffer (25 mM Tris, 192 mM Glycine, 0.1 % SDS) for 15 min to ensure blotting of proteins on PVDF membranes using the Trans-Blot Turbo transfer system (Bio-Rad, USA). Membrane was then blocked using 5 % milk in 0.01 % Tween in tris-buffered saline (TBS-T) buffer (5 mM Tris, 15 mM NaCl) followed by incubation in primary antibody (syn-1 1:2,000, BD Biosciences, UK; 11A5 1:5,000, Prothena Biosciences Inc.) in 2 % milk in TBS-T at 4 °C over night. After washing membranes 3x 10min with TBS-T, secondary goat anti mouse antibody (Santa Cruz, USA) was incubated at 1:10,000 in 2 % milk in TBS-T for 1 h. Membranes were rinsed with TBS-T, incubated in an ECL mix of 1:1 peroxide to luminol solution (Clarity Western ECL Substrate, Bio-Rad, USA) and placed into the ChemiDoc Imaging system (Bio-Rad, USA).

#### For m-asyn

The expression and purification was carried out as described previously [46]. Briefly, bacterial expression vector pRK172 containing m-asyn gene (kindly provided by Virginia Lee’s laboratory, Institute on Aging and Center for Neurodegenerative Disease Research, University of Pennsylvania, Philadelphia, USA) was inserted into E.coli BL-21 bacteria for protein expression and grown using TB media. Spun down bacterial pellets were then re-suspended in high-salt lysis buffer (750 mM NaCl, 100 mM MES, 1 mM EDTA, pH 7.0) containing protease inhibitors. The lysate was then heated to 100 °C for 10 min and centrifuged at 10,000×g for 30 min. The supernatant was dialyzed against 10 mM Tris at pH 7.5 and applied first to a size exclusion column (Superdex 200) followed by a Mono Q column.

#### For S129A h-asyn

The pT7-7 vector containing the S129A h-asyn gene was kindly provided by Hilal Lashuel’s laboratory (Brain Mind Institute, Ecole Polytechnique Federale de Lausanne). The vector was inserted in E-Coli BL-21 bacteria and grown in LB media. The bacterial lysate was subjected to gel filtration purification using Superdex 200 chromatography column, followed by anion exchange purification by Mono-Q column.

### Animals

C57/Bl6-OlaHsd mice - lacking the asyn gene - were obtained from Harlan (Kentucky, USA) and young adult female Sprague Dawley rats (200–225 g) obtained from Charles River (Kisslegg, Germany). Animals were housed with ad libitum *access* to food and water on a 12 h light-dark cycle. All experimental procedures were approved by the Ethical Committee for Use of Laboratory Animals in the Lund-Malmö region.

Rat brain samples overexpressing h-asyn were obtained in a separate study (Landeck et al., unpublished data). Briefly, neonatal Sprague Dawley rat pups were injected with rAAV5 vectors expressing h-asyn into the dorsal striatum. Twelve months after injection, frontal brain area was dissected and stored at -80 °C for further analysis. Samples were used for evaluation of antibodies on western blot and assay screening only.

### Preparation of tissue matrix used for Western blot and recombinant protein spiking

Brain tissue samples for WB analysis and recombinant protein spiking were extracted using a ratio of 1:5 weight per volume of Tris lysis buffer (144 mM NaCl, 50 mM Tris, 2 mM EDTA, pH 7.6) or Tris buffer containing 1 % SDS. Protease and Phosphatase inhibitors (Roche, Switzerland) were added to buffers before use. Tissue was lysed using a probe sonicator (Sonics & materials, USA) 10× 2 s at 40 Hz, incubated on ice for 15 min and centrifuged for 10 min at 21,000 rcf at 4 °C. Supernatant was transferred to a new tube. Protein concentration was determined using the DC protein assay kit (Bio-Rad, USA) and samples were stored at -80 °C.

### Western blot analysis for determination of antibody specificity

Protein lysate (50 μg for asyn specific antibodies or 100 μg pS129 asyn specific antibodies) and recombinant protein (2 ng) were separated on SDS-polyacrylamide gels with an 8–16 % gradient (Bio-Rad, USA) and transferred onto PVDF membranes using the Trans-Blot Turbo transfer system (Bio-Rad, USA). The membranes were incubated with 5 % milk in TBS-T buffer for 1 h at room temperature (RT) to block unspecific binding. Primary antibodies were added at a concentration of 0.2 μg/ml for asyn specific antibodies, 1 μg/ml for pS129 asyn specific antibodies or 1:200 dilution for EP1536Y and pSyn#64 in 2 % milk in TBS-T and incubated over night at 4 °C. The next day the membranes were washed with TBS-T and the appropriate peroxidase-coupled secondary antibody (Santa Cruz, USA) at 1:10,000 was added in 2 % milk in TBS-T for 1 h. After rinsing with TBS-T the membranes were incubated in an ECL mix of 1:1 peroxide to luminol solution (Clarity Western ECL Substrate, Bio-Rad, USA) and placed into the ChemiDoc Imaging system (Bio-Rad, USA).

### HEK293 cell transfection and lysis

PEI solution was prepared by dissolving Polyethyleneimine (Polysciences, USA) using 80 °C warm H_2_O to reach a concentration of 1 g/l. Solution was let cool to RT, pH was set to 7.0 by adding HCl, H_2_O was added to reach final concentration and solution was filtered sterile using a 0.22 μm membrane. Aliquots were stored at -20 °C. On the day of transfection PEI solution was thawed in a 37 °C water bath. 1 μg DNA in total and 3 μg PEI (added last) in 100 μl were used per ml culture medium, mixed by vortexing, incubated at RT for 15 min and added to cell culture at a confluency of 75–90 % on a 6-well plate. 48 h later cells were collected in 1 ml of PBS, transferred to a tube and spun down at 200 rcf for 5 min. Supernatant was discarded and 100 μl of lysis buffer [1 % SDS, 144 mM NaCl, 50 mM Tris, 2 mM EDTA, pH 7.6, protease and phosphatase inhibitors (Roche, Switzerland)] was added to the cell pellet. Cells were then sonicated (10× 2 s at 40Hz), the solution was incubated on ice for 15 min and then centrifuged at 21,000×g for 15 min at 4 °C. Supernatant was transferred to a new tube, protein concentration determined using the DC protein assay kit (Bio-Rad, USA) and samples stored at -80 °C. Samples were diluted 1:10 in PBS before run on the assay.

### Production of viral vectors

Recombinant adeno-associated viral vectors serotype 6 (rAAV6) contained a synapsin1 promoter (syn1) expressing either h-asyn, polo-like kinase 2 (PLK2), a PLK2 dead mutant (mPLK2) or GFP followed by a h-SV40 polyA sequence. These cassettes were flanked by inverted terminal repeats (ITR2). HEK293 cells at 70–80 % confluence were co-transfected using the calcium-phosphate precipitation method including the rAAV plasmid and helper as previously described [47]. After 3 days of incubation, cells were harvested and lysed by performing three freeze-thaw cycles in a dry ice/ethanol bath. After treatment with benzonase (Sigma-Aldrich, Sweden) the lysate was purified using a discontinuous iodixanol gradient followed by Separose Q column chromatography [48] and then concentrated with a 100kD cut-off column (Millipore Amicon Ultra, Millipore, Sweden). To determine the titer of the viral vector solutions quantitative PCR with probes targeting the ITR sequence was performed. Prior to use in experiments, vectors were mixed 1:1 and diluted using PBS to a concentration of 5E13 gc/ml per vector (1E14 gc/ml per vector mix) and re-titered.

### Animals and stereotaxic surgery

Young adult Sprague Dawley rats were anesthetized by i.p. injection of 6 ml/kg of a 20:1 mixture of Fentanyl and Dormitor (Apoteksbolaget, Sweden). After placing the animal into a stereotaxic frame (Stoelting, USA), 2 μl of rAAV6 vector solution was injected into the right SN using the following coordinates: anteroposterior (AP) -5.0 mm, mediolateral (ML) -2.0 mm from bregma and dorsoventral (DV) -7.2 mm from the dura. The tooth bar was adjusted to -2.3 mm. 2 μl of vector was deposited into the left striatum using the following coordinates: AP +1.0 mm, ML +3.0 mm from bregma and DV -5.0 mm from the dura. The tooth bar was adjusted to 0.0 mm. Injection was performed using a pulled and cut glass capillary (final outer diameter of about 60–80 μm) attached onto a 5 μl Hamilton syringe with a 22 s gauge needle. After delivery of the viral vector using a pulsed injection of 0.1 μl every 15 s, the capillary was held in place for 5 min, retracted 0.2 mm and after 1 min it was slowly withdrawn from the brain. Temgesic and Antisedan (Apoteksbolaget, Sweden) were administered s.c. as analgesic treatment and to reverse anesthesia, respectively.

### Rat brain tissue lysis

Dissected and fresh frozen brain tissue samples were sequentially lysed 200 mg/ml in first Triton lysis buffer [150 mM NaCl, 50 mM Tris pH 7.6, 1 % Triton X-100, 2 mM EDTA containing protease and phosphatase inhibitors (Roche, Switzerland)] by sonication 15× for 1 s. Ipsilateral striatum to ventral midbrain injected brains were sequentially lysed in first 20 mM Tris-acetate buffer (TAE) pH 6.1, sonicated 15× for 1 s, 80 μl of each brain lysate were transferred to a new tube and 9 μl of 10 % Triton containing 10× protease and phosphatase inhibitors added. After incubation for 10 min on ice, all samples were centrifuged at 21,000 rcf for 10 min at 4 °C and supernatant transferred to new tubes. Lysing step was repeated two more times, supernatant from all lysing steps for each brain were collected in one single tube, termed here Triton fraction. Leftover pellet was lysed in SDS lysis buffer (150 mM NaCl, 50 mM Tris pH 7.6, 1 % SDS, 2 mM EDTA containing protease and phosphatase inhibitors) twice by repeating steps described above at RT. Both supernatants were combined in one tube for each brain, termed here the SDS fraction and contain a fraction of proteins in the samples that are either resistant to Triton solubilization or located within the nucleus. Protein concentration of each fraction was determined using the DC™ protein assay kit (Bio-Rad, USA). Triton fractions were diluted 1:300 for ventral midbrain and 1:100 for striatal samples before running them on the AlphaLISA duplex assay while SDS fractions were diluted 1:10.

### Rat brain histological analysis

Animals were killed at 2 weeks after vector treatment by sodium pentobarbital (Apoteksbolaget, Sweden) and perfused with 50 ml of 0.9 % NaCl followed by 250 ml of ice-cold 4 % paraformaldehyde (PFA in 0.1 M phosphate buffer, pH7.4) for 5 min. Brains were removed, post-fixed in 4 % PFA for 24 h and then transferred into 25 % sucrose. Then brains were cut using a semi-automated freezing microtome (Microm HM 450) into 35 μm thick coronal sections and stored in antifreeze solution (0.5 M phosphate buffer, 30 % glycerol, 30 % ethylene glycol) at -20 °C.

Histological processing was performed using an immunohistochemical staining protocol on free-floating sections. Brain sections were washed using TBS. To quench the endogenous peroxidase activity sections were incubated in 3 % H_2_O_2_ and 10 % methanol in TBS buffer for 30 min. After 3 washes with TBS buffer, sections were incubated for 30 min in 0.05 % Triton X-100 in TBS buffer (TBS-T) containing 5 % normal serum from the same species the secondary antibody was raised in. The primary antibody asyn211 (1:100,000) was applied in 1 % BSA in TBS-T over night at RT. The next day sections were rinsed with TBS-T and incubated with horse anti mouse biotinylated secondary antibody (1:200, Vector Laboratories Inc.) in 1 % BSA in TBS-T for 1 h. Sections were again washed with TBS-T and incubated with an avidin-biotin-peroxidase complex solution (Vectastain ABC kit, Vector Laboratories Inc., USA) for 1 h. The specific staining was visualized using 3,3′-diaminobenzidine (DAB Safe, Saveen Werner, Sweden) and 0.01 % H_2_O_2_. For preservation and visualization, sections were mounted on chromatin-gelatin coated glass slides, dried, dehydrated in increasing alcohol solutions, cleared in xylene and coverslipped using DPX (Sigma-Aldrich, Sweden).

For double labeling of h-asyn and PLK2, sections were washed using KPBS (3.4 mM KH_2_PO_4_, 15.3 mM K_2_HPO_4_, 145 mM NaCl) and incubated in Tris/EDTA buffer (10 mM Tris, 1 mM EDTA, pH 9) at 80 °C for 30 min. After letting the sections cool down for 20 min, specimens were washed with KPBS and incubated in 0.25 % Triton in KPBS containing 5 % normal serum from same species the secondary antibody was raised in. Then, the primary antibodies asyn211 (1:10,000) and PLK2 (1:200, sc-25421, Santa Cruz, USA) were added and incubated over night at RT. The next day sections were rinsed with KPBS and incubated in 0.25 % Triton + 5 % normal serum in KPBS for 1 h containing secondary antibody biotinylated goat anti rabbit (1:200, Vector Laboratories Inc., USA). Sections were again washed with KPBS and incubated with 0.25 % triton + 5 % normal serum in KPBS containing AlexaFluor647 conjugated streptavidin (1:200, Invitrogen, USA) and secondary antibody donkey anti mouse coupled to AlexaFluor488 fluorophore (Jackson, USA) for 2 h. After washes with KPBS, sections were mounted and coverslipped using PVA-DABCO mounting medium (Sigma-Aldrich, Sweden). Confocal images were taken using a Nikon eclipse Ti inverted microscope with a 60× oil lens and the Nikon D-eclipse C1 detector.

For cresyl violet staining, sections were mounted on chromatin-gelatin coated glass slides and dried overnight, hydrated in decreasing alcohol solutions and stained for 30 s in 0.5 % cresyl violet (Sigma-Aldrich, Sweden) + 0.1 % acidic acid. After a wash in H_2_O, sections were dehydrated in increasing alcohol solutions, cleared in xylene and coverslipped using DPX.

### Densitometry

Staining intensity of DARPP32- and VMAT2-possitive neurons was measured on digital images taken from coronal striatal and ventral midbrain sections, respectively, using the Zeiss microscope (Axio Zoom.V16, Zeiss, Germany). Every 24^th^ section of the striatum and every 12^th^ section of the ventral midbrain in the rostro-caudal axis was outlined using ImageJ and optical density readings were corrected for non-specific background using density measurements from the corpus callosum or dorsal midbrain of each animal.

### Human tissue processing and analysis

Human brain tissues were obtained from the Sydney Brain Bank at Neuroscience Research Australia. Standardized clinicopathological criteria were used for diagnosis [49] with dementia status and severity determined using the Clinical Dementia Rating (CDR) scale [50]. Parkinson’s disease (PD) cases fulfilled the UK PD Society Brain Bank Diagnostics Criteria [51] and did not have any mutations correlated with PD. Controls (Ctrl) had no significant neuropathology and no evidence of neurological or psychiatric disease. Ethics approval for this study was obtained from the Human Research Ethics Committee at the University of New South Wales. Demographic details for each group are listed as median (interquartile range): The age range of cases (*n* = 16) used for assay reproducibility tests was 74 (22) years, for Ctrl group (*n* = 8) was 90 (6) years, for PD group (*n* = 8) was 82 (10.5) years and for PDD group (*n* = 8) was 81 (7) years. The onset as well as duration of PD was 64 (9) and 17 (6) years for PD group, and 67 (8) and 15 (3) years for PDD group, respectively. The duration of dementia for the PDD group was 3.5 (2) years. Post-mortem interval for the cases used for reproducibility testing was 29 (19) hours, for Ctrl, PD and PDD groups 23 (11), 18 (18) and 24 (23) hours.

Tissue samples from the Ctrl, PD and PDD cohorts were homogenized 200 mg/ml in 20 mM Tris-acetate (TAE) buffer pH 6.1 using stainless steel beads and a Tissuelyser II system (Qiagen) at 20 Hz for 40 s. Enzymatic analysis of the same cohort of samples was performed earlier [42]. Lysate (150 μl) was transferred to a new tube, 16 μl of 10 % Triton containing 10× protease and phosphatase inhibitors (Roche, Switzerland) added (for sequential extraction) and samples sonicated 10× 1 s at 40Hz. Tissue samples used for the reproducibility testing were lysed in 20 mM TAE buffer pH 6.1 and sonicated 10x for 1 s at 40 Hz. After incubation on ice for 10 min and centrifugation for 10 min at 21,000 rcf and 4 °C, supernatant was transferred to a new tube and lysing step repeated twice more using 60 μl 1 % Triton in TAE buffer + inhibitors, supernatants were combined to a single tube, here referred to as Triton fraction. Remaining pellet was again lysed in two consecutive steps (as described above at RT) in 75 μl of 1 % SDS in TAE buffer + inhibitors, both supernatants were combined to a single tube, termed here the SDS fraction. Protein concentration of each fraction was determined using the DC™ protein assay kit (Bio-Rad, USA). Triton fractions were diluted 1:300 before running samples on the AlphaLISA duplex assay while SDS fractions were diluted 1:10.

### Data processing and statistical analysis

S/B ratio was calculated by dividing the ‘signal of analyte’ by the ‘signal of blank’. Hook-point values were calculated by subtracting the background signal from the positive sample signal. The LDL was calculated as mean of 9 blank replicates + 3*SD. Standard curve fitting was done using the GraphPad Prism6 program. A sigmoid weighted curve was fitted on signal measured and corresponding log transformed asyn concentration values. A fitting of R^2^ ≥90.5 % was accepted. Blanks were set to 1E-25 mg/ml. Intra and inter assay variations were determined by calculating the concentration of a sample containing a known amount of h-asyn using the fitted model of a standard curve run on the same plate. Relative standard deviations of analyzed concentration values were calculated for matching samples run on the same day or on different days. Sample reproducibility was assessed by repeated measures for each of the 16 cases over several days. Each repeated value is expressed as percent of mean of all repeats. Z’ values were calculated using the following equation:

$$ \mathrm{Z}'=1\hbox{--} \left|\frac{3\times \left(\mathrm{S}\mathrm{T}\mathrm{D}\ {\mathrm{signal}}_{\mathrm{analyte}}\hbox{--} \mathrm{S}\mathrm{T}\mathrm{D}\ {\mathrm{signal}}_{\mathrm{blank}}\right)}{\mathrm{mean}\ {\mathrm{signal}}_{\mathrm{analyte}}\hbox{--} \mathrm{mean}\ {\mathrm{signal}}_{\mathrm{blank}}}\right| $$

Data is shown as mean ± standard error of the mean (SEM). Statistical analysis was carried out using the GraphPad Prism6 program (GraphPad Software, La Jolla, CA, USA). For HEK293 and rat brain measurements, a two-tailed Mann-Whitney test was used. Human data sets were first tested for normal distribution using the D’Agostino & Pearson omnibus normality test. Then either a one-way ANOVA followed by a Tukey’s HSD test for normally distributed measurements or a Kruskal-Wallis followed by a Dunn’s multiple comparisons test was used. Differences were considered significant for *p* <0.05.

## Abbreviations

11A5-Eu, 11A5 antibody coupled to Europium Acceptor-beads; asyn, alpha-synuclein; AU, arbitrary units; CSF, cerebrospinal fluid; Ctrl, control; h-asyn, human alpha-synuclein; k.o., knock out; LB509-Tb, LB509 antibody coupled to Terbium Acceptor-beads; LBs, Lewy bodies; LDL, lower detection limit; m-asyn, mouse alpha-synuclein; mPLK2, Polo-like kinase 2, dead mutant; PD, Parkinson’s disease; PDD, Parkinson’s disease with dementia; PLK2; Polo-like kinase 2; pS129, phosphorylated at serine 129; rAAV6, recombinant adeno-associated virus serotype 6; S/B, signal to background; SN, substantia nigra; uninj., uninjected; WT, wild type

## Additional files

Additional file 1: Figure S1. Principle and protocol of the AlphaLISA technique. In **A** Streptavidin-coated Donor-beads are bound to biotinylated antibodies. Acceptor-beads are directly coupled to antibodies. When both antibodies bind to an analyte, the beads are brought into close proximity. Donor-beads are excited at 680 nm and produce singlet oxygen molecules triggering a chemical reaction in the close-by Europium Acceptor-beads resulting in an emission of light at 615 nm. In **B** the protocol of a no-wash AlphaLISA is depicted. (PDF 315 kb) Additional file 2: Figure S2. Quantification of S129 phosphorylation of human alpha-synuclein recombinant protein. An isoelectric focusing gel pH range 5–8 **(A)** was used to separate pS129 from non-phosphorylated h-asyn by loading recombinant h-asyn and pS129 h-asyn protein side by side. In **B**, blots were probed for total h-asyn (syn-1) or pS129 h-asyn (11A5) to determine pS129 h-asyn band. Quantification areas are indicated on total h-asyn blots by dashed boxes. This assay was run independently on three occasions, which yielded estimates of percent phosphorylation of h-asyn at S129 site to be 28.4, 29.5, and 26.0 %, respectively, with an average and standard deviation of 28.0 ± 1.8 %. (PDF 339 kb) Additional file 3: Figure S3. Scheme of AlphaLISA duplex assay for total and S129 phosphorylated human alpha-synuclein detection. The 4B12 antibody – binding to aa 103–108 – is biotinylated and used to bind the Streptavidin-coated Donor-beads. Terbium Acceptor-beads were coupled to LB509 antibodies for total h-asyn quantification while the 11A5 antibody was used and attached to Europium Acceptor-beads in order to measure pS129 h-asyn. Both antibodies recognize an epitope close to each other (aa 115–122 and aa 129 respectively). (PDF 500 kb) Additional file 4: Figure S4. Principle of steric hindrance analysis and filter bleed-through. A hypothetical illustration of signal change caused by a possible steric hindrance of 11A5-Europium (11A5-Eu) and LB509-Terbium (LB509-Tb) molecules when being added together in one well is depicted in **A**. At baseline **(A1)** no steric hindrance is possible since only one Acceptor-bead coupled antibody is in solution, here shown for Terbium Acceptor-beads. Adding the second antibody (in this case 11A5) coupled to Europium Acceptor-beads can either result in signal reduction **(A2)** or in similar emission intensity **(A3)**. Hypothetical signal values are illustrated in **A**’. Filter bleed-through for this assay protocol was determined by four measurement conditions illustrated as examples in **B1**-**B4**. First background signal for Terbium Acceptor-bead was established by using the appropriate filter as well as by not adding any analyte or Europium Acceptor-bead **(B1)**. Background signal for Europium filter was measured by using the same well **(B3)**. Adding the analyte to the above described mix will result in a corresponding signal when the Terbium filter is used **(B2)** and in a false signal (X % of Terbium signal) when the Europium filter is used **(B4)**. Example of signals (green for Terbium, red for Europium) and % calculations is illustrated in **B**’. Same measurements and calculations were done for the Terbium assay protocol. (PDF 528 kb) Additional file 5: Figure S5. Assessment of optimal Donor-bead concentration. The ideal Donor-bead concentration was determined once simultaneously in a mix of Europium Acceptor-bead coupled 11A5 antibody and Terbium Acceptor-bead coupled LB509 antibody. Arrows indicate optimal 4B12 concentrations for both Acceptor-bead variants. AU: arbitrary units. (PDF 156 kb) Additional file 5: Figure S6. Assessment of assay reproducibility in the duplex assay using cortical human samples. Tissue samples of the frontal cortex (*n* = 16) were lysed using buffer containing 1 % Triton and repeatedly analyzed (*n* = 5) using the duplex assay for total and pS129 h-asyn concentration on separate days. Percent variation from the mean value for each sample is plotted for total h-asyn measurements (**A**), for pS129 h-asyn (**B**) and for total to pS129 h-asyn ratio (**C**). Dashed lines indicate ± 25 % variation while thin lines indicate ± 10 % variation from mean (0 %). (PDF 233 kb) Additional file 7: Figure S7. Endogenous rat total and S129 phosphorylated levels. Fresh frozen striatal and ventral midbrain tissue samples of naïve rats were sequentially lysed in first 1 % Triton **(A)** then in 1 % SDS containing lysis buffer **(B)** and diluted 1:300 or 1:10, respectively. Samples (*n* = 6 per region) were run on AlphaLISA uniplex assays using the biotinylated syn-1 antibody (BD Biosciences, UK) combined with either 4D6 (Abcam, UK) or 11A5 (Prothena Biosciences Inc.) conjugated on the Europium Acceptor-beads for the detection of total and pS129 asyn, respectively. As reference protein, pS129 h-asyn was used for both assays and spiked into appropriate asyn knockout mouse brain lysate. Total (open) and pS129 (filled) r-asyn levels are presented in overlapping bars. Numbers inside the bars show the percent of pS129 to total r-asyn. Error bars indicate SEM. (PDF 173 kb)

## References

[1] Tanner CM (1996). Early intervention in Parkinson’s disease: epidemiologic considerations. Ann Epidemiol.

[2] Langston JW (1998). Epidemiology versus genetics in Parkinson’s disease: progress in resolving an age-old debate. Ann Neurol.

[3] Bernheimer H, Birkmayer W, Hornykiewicz O, Jellinger K, Seitelberger F (1973). Brain dopamine and the syndromes of Parkinson and Huntington. Clinical, morphological and neurochemical correlations. J Neurol Sci.

[4] Whone AL, Watts RL, Stoessl AJ, Davis M, Reske S, Nahmias C, Lang AE, Rascol O, Ribeiro MJ, Remy P (2003). Slower progression of Parkinson’s disease with ropinirole versus levodopa: the REAL-PET study. Ann Neurol.

[5] McGeer PL, Itagaki S, Boyes BE, McGeer EG (1988). Reactive microglia are positive for HLA-DR in the substantia nigra of Parkinson’s and Alzheimer’s disease brains. Neurology.

[6] Orth M, Schapira AH (2002). Mitochondrial involvement in Parkinson’s disease. Neurochem Int.

[7] Jenner P (2003). Oxidative stress in Parkinson’s disease. Ann Neurol.

[8] Chu Y, Dodiya H, Aebischer P, Olanow CW, Kordower JH (2009). Alterations in lysosomal and proteasomal markers in Parkinson’s disease: relationship to alpha-synuclein inclusions. Neurobiol Dis.

[9] Dickson DW, Fujishiro H, Orr C, DelleDonne A, Josephs KA, Frigerio R, Burnett M, Parisi JE, Klos KJ, Ahlskog JE (2009). Neuropathology of non-motor features of Parkinson disease. Parkinsonism Relat Disord.

[10] Wolters E (2009). Non-motor extranigral signs and symptoms in Parkinson’s disease. Parkinsonism Relat Disord.

[11] Spillantini MG, Crowther RA, Jakes R, Hasegawa M, Goedert M (1998). alpha-synuclein in filamentous inclusions of Lewy bodies from Parkinson’s disease and dementia with Lewy bodies. Proc Natl Acad Sci U S A.

[12] Baba M, Nakajo S, Tu PH, Tomita T, Nakaya K, Lee VM, Trojanowski JQ, Iwatsubo T (1998). Aggregation of alpha-synuclein in Lewy bodies of sporadic Parkinson’s disease and dementia with Lewy bodies. Am J Pathol.

[13] Giasson BI, Duda JE, Murray IV, Chen Q, Souza JM, Hurtig HI, Ischiropoulos H, Trojanowski JQ, Lee VM (2000). Oxidative damage linked to neurodegeneration by selective alpha-synuclein nitration in synucleinopathy lesions. Science.

[14] Fujiwara H, Hasegawa M, Dohmae N, Kawashima A, Masliah E, Goldberg MS, Shen J, Takio K, Iwatsubo T (2002). alpha-Synuclein is phosphorylated in synucleinopathy lesions. Nat Cell Biol.

[15] Anderson JP, Walker DE, Goldstein JM, de Laat R, Banducci K, Caccavello RJ, Barbour R, Huang J, Kling K, Lee M (2006). Phosphorylation of Ser-129 is the dominant pathological modification of alpha-synuclein in familial and sporadic Lewy body disease. J Biol Chem.

[16] El-Agnaf OM, Salem SA, Paleologou KE, Curran MD, Gibson MJ, Court JA, Schlossmacher MG, Allsop D (2006). Detection of oligomeric forms of alpha-synuclein protein in human plasma as a potential biomarker for Parkinson’s disease. FASEB J.

[17] Mollenhauer B, Cullen V, Kahn I, Krastins B, Outeiro TF, Pepivani I, Ng J, Schulz-Schaeffer W, Kretzschmar HA, McLean PJ (2008). Direct quantification of CSF alpha-synuclein by ELISA and first cross-sectional study in patients with neurodegeneration. Exp Neurol.

[18] Hong Z, Shi M, Chung KA, Quinn JF, Peskind ER, Galasko D, Jankovic J, Zabetian CP, Leverenz JB, Baird G (2010). DJ-1 and alpha-synuclein in human cerebrospinal fluid as biomarkers of Parkinson’s disease. Brain.

[19] Emmanouilidou E, Elenis D, Papasilekas T, Stranjalis G, Gerozissis K, Ioannou PC, Vekrellis K (2011). Assessment of alpha-synuclein secretion in mouse and human brain parenchyma. PLoS One.

[20] Bidinosti M, Shimshek DR, Mollenhauer B, Marcellin D, Schweizer T, Lotz GP, Schlossmacher MG, Weiss A (2012). Novel one-step immunoassays to quantify alpha-synuclein: applications for biomarker development and high-throughput screening. J Biol Chem.

[21] Kruse N, Persson S, Alcolea D, Bahl JM, Baldeiras I, Capello E, Chiasserini D, Bocchio Chiavetto L, Emersic A, Engelborghs S (2015). Validation of a quantitative cerebrospinal fluid alpha-synuclein assay in a European-wide interlaboratory study. Neurobiol Aging.

[22] Majbour NK, et al. "Oligomeric and phosphorylated alpha-synuclein as potential CSF biomarkers for Parkinson’s disease." Mol Neurodegener. 2016;11:7. 10.1186/s13024-016-0072-9 PMC4717559 26782965

[23] Foulds PG, Mitchell JD, Parker A, Turner R, Green G, Diggle P, Hasegawa M, Taylor M, Mann D, Allsop D (2011). Phosphorylated alpha-synuclein can be detected in blood plasma and is potentially a useful biomarker for Parkinson’s disease. FASEB J.

[24] Wang Y, Shi M, Chung KA, Zabetian CP, Leverenz JB, Berg D, Srulijes K, Trojanowski JQ, Lee VM, Siderowf AD (2012). Phosphorylated alpha-synuclein in Parkinson’s disease. Sci Transl Med.

[25] Swirski M, Miners JS, de Silva R, Lashley T, Ling H, Holton J, Revesz T, Love S (2014). Evaluating the relationship between amyloid-beta and alpha-synuclein phosphorylated at Ser129 in dementia with Lewy bodies and Parkinson’s disease. Alzheimers Res Ther.

[26] Mbefo MK, Paleologou KE, Boucharaba A, Oueslati A, Schell H, Fournier M, Olschewski D, Yin G, Zweckstetter M, Masliah E (2010). Phosphorylation of synucleins by members of the Polo-like kinase family. J Biol Chem.

[27] Tokuda T, Salem SA, Allsop D, Mizuno T, Nakagawa M, Qureshi MM, Locascio JJ, Schlossmacher MG, El-Agnaf OM (2006). Decreased alpha-synuclein in cerebrospinal fluid of aged individuals and subjects with Parkinson’s disease. Biochem Biophys Res Commun.

[28] Tinsley RB, Kotschet K, Modesto D, Ng H, Wang Y, Nagley P, Shaw G, Horne MK (2010). Sensitive and specific detection of alpha-synuclein in human plasma. J Neurosci Res.

[29] Inglis KJ, Chereau D, Brigham EF, Chiou SS, Schobel S, Frigon NL, Yu M, Caccavello RJ, Nelson S, Motter R (2009). Polo-like kinase 2 (PLK2) phosphorylates alpha-synuclein at serine 129 in central nervous system. J Biol Chem.

[30] Bergeron M, Motter R, Tanaka P, Fauss D, Babcock M, Chiou SS, Nelson S, San Pablo F, Anderson JP (2014). In vivo modulation of polo-like kinases supports a key role for PLK2 in Ser129 alpha-synuclein phosphorylation in mouse brain. Neuroscience.

[31] Foulds PG, Yokota O, Thurston A, Davidson Y, Ahmed Z, Holton J, Thompson JC, Akiyama H, Arai T, Hasegawa M (2012). Post mortem cerebrospinal fluid alpha-synuclein levels are raised in multiple system atrophy and distinguish this from the other alpha-synucleinopathies, Parkinson’s disease and Dementia with Lewy bodies. Neurobiol Dis.

[32] Foulds PG, Diggle P, Mitchell JD, Parker A, Hasegawa M, Masuda-Suzukake M, Mann DM, Allsop D (2013). A longitudinal study on alpha-synuclein in blood plasma as a biomarker for Parkinson’s disease. Sci Rep.

[33] Stewart T, Sossi V, Aasly JO, Wszolek ZK, Uitti RJ, Hasegawa K, Yokoyama T, Zabetian CP, Leverenz JB, Stoessl AJ (2015). Phosphorylated alpha-synuclein in Parkinson’s disease: correlation depends on disease severity. Acta Neuropathol Commun.

[34] Mollenhauer B, El-Agnaf OM, Marcus K, Trenkwalder C, Schlossmacher MG (2010). Quantification of alpha-synuclein in cerebrospinal fluid as a biomarker candidate: review of the literature and considerations for future studies. Biomark Med.

[35] Salvi M, Trashi E, Marin O, Negro A, Sarno S, Pinna LA (2012). Superiority of PLK-2 as alpha-synuclein phosphorylating agent relies on unique specificity determinants. Biochem Biophys Res Commun.

[36] Oueslati A, Schneider BL, Aebischer P, Lashuel HA (2013). Polo-like kinase 2 regulates selective autophagic alpha-synuclein clearance and suppresses its toxicity in vivo. Proc Natl Acad Sci U S A.

[37] Braak H, Del Tredici K, Rub U, de Vos RA, Jansen Steur EN, Braak E (2003). Staging of brain pathology related to sporadic Parkinson’s disease. Neurobiol Aging.

[38] Burre J, Sharma M, Sudhof TC (2014). alpha-Synuclein assembles into higher-order multimers upon membrane binding to promote SNARE complex formation. Proc Natl Acad Sci U S A.

[39] Wang L, Das U, Scott DA, Tang Y, McLean PJ, Roy S (2014). alpha-synuclein multimers cluster synaptic vesicles and attenuate recycling. Curr Biol.

[40] Tong J, Wong H, Guttman M, Ang LC, Forno LS, Shimadzu M, Rajput AH, Muenter MD, Kish SJ, Hornykiewicz O, Furukawa Y (2010). Brain alpha-synuclein accumulation in multiple system atrophy, Parkinson’s disease and progressive supranuclear palsy: a comparative investigation. Brain.

[41] Zhou J, Broe M, Huang Y, Anderson JP, Gai WP, Milward EA, Porritt M, Howells D, Hughes AJ, Wang X, Halliday GM (2011). Changes in the solubility and phosphorylation of alpha-synuclein over the course of Parkinson’s disease. Acta Neuropathol.

[42] Hall H, Reyes S, Landeck N, Bye C, Leanza G, Double K, Thompson L, Halliday G, Kirik D (2014). Hippocampal Lewy pathology and cholinergic dysfunction are associated with dementia in Parkinson’s disease. Brain.

[43] Bielefeld-Sevigny, M. "AlphaLISA immunoassay platform- the "no-wash" high-throughput alternative to ELISA." Assay Drug Dev Technol. 2009;7(1):90-92. 10.1089/adt.2009.9996 19382891

[44] Lu JH, Ardah MT, Durairajan SS, Liu LF, Xie LX, Fong WF, Hasan MY, Huang JD, El-Agnaf OM, Li M (2011). Baicalein inhibits formation of alpha-synuclein oligomers within living cells and prevents Abeta peptide fibrillation and oligomerisation. Chembiochem.

[45] Ardah MT, Paleologou KE, Lv G, Abul Khair SB, Kazim AS, Minhas ST, Al-Tel TH, Al-Hayani AA, Haque ME, Eliezer D, El-Agnaf OM (2014). Structure activity relationship of phenolic acid inhibitors of alpha-synuclein fibril formation and toxicity. Front Aging Neurosci.

[46] Luk KC, Kehm V, Carroll J, Zhang B, O’Brien P, Trojanowski JQ, Lee VM (2012). Pathological alpha-synuclein transmission initiates Parkinson-like neurodegeneration in nontransgenic mice. Science.

[47] Grimm D, Kern A, Rittner K, Kleinschmidt JA (1998). Novel tools for production and purification of recombinant adenoassociated virus vectors. Hum Gene Ther.

[48] Zolotukhin S, Byrne BJ, Mason E, Zolotukhin I, Potter M, Chesnut K, Summerford C, Samulski RJ, Muzyczka N (1999). Recombinant adeno-associated virus purification using novel methods improves infectious titer and yield. Gene Ther.

[49] Halliday G, Ng T, Rodriguez M, Harding A, Blumbergs P, Evans W, Fabian V, Fryer J, Gonzales M, Harper C (2002). Consensus neuropathological diagnosis of common dementia syndromes: testing and standardising the use of multiple diagnostic criteria. Acta Neuropathol.

[50] Morris JC (1997). Clinical dementia rating: a reliable and valid diagnostic and staging measure for dementia of the Alzheimer type. Int Psychogeriatr.

[51] Hughes AJ, Daniel SE, Kilford L, Lees AJ (1992). Accuracy of clinical diagnosis of idiopathic Parkinson’s disease: a clinico-pathological study of 100 cases. J Neurol Neurosurg Psychiatry.

