# Supplementary material for: A novel multiplex assay for simultaneous quantification of total and S129 phosphorylated human alpha-synuclein
Source: Mol Neurodegener. 2016 Aug 22;11(1):61. doi: 10.1186/s13024-016-0125-0 (PMC4994244; doi:10.1186/s13024-016-0125-0)
Supplement: Additional file 1: Figure S1. — Principle and protocol of the AlphaLISA technique. In A Streptavidin-coated Donor-beads are bound to biotinylated antibodies. Acceptor-beads are directly coupled to antibodies. When both antibodies bind to an analyte, the beads are brought into close proximity. Donor-beads are excited at 680 nm and produce singlet oxygen molecules triggering a chemical reaction in the close-by Europium Acceptor-beads resulting in an emission of light at 615 nm. In B the protocol of a no-wash AlphaLISA is depicted. (PDF 315 kb) [file 13024_2016_125_MOESM1_ESM.pdf]

 DOI 10.1186/s13024-016-0125-0

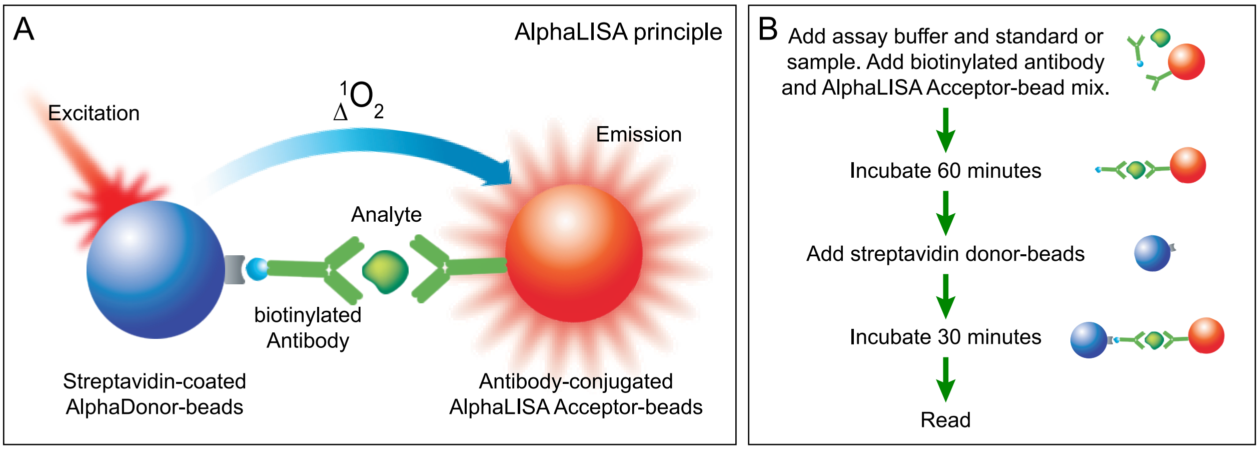

© 2009-2016 PerkinElmer, Inc. All rights reserved. Printed with permission.

Additional file 1: Figure S1. *Principle and protocol of the AlphaLISA technique.* In **A** Streptavidin-coated Donor-beads are bound to biotinylated antibodies. Acceptor-beads are directly coupled to antibodies. When both antibodies bind to an analyte, the beads are brought into close proximity. Donor-beads are excited at 680nm and produce singlet oxygen molecules triggering a chemical reaction in the close-by Europium Acceptor-beads resulting in an emission of light at 615nm. In **B** the protocol of a no-wash AlphaLISA is depicted.
