# Supplementary material for: A novel multiplex assay for simultaneous quantification of total and S129 phosphorylated human alpha-synuclein
Source: Mol Neurodegener. 2016 Aug 22;11(1):61. doi: 10.1186/s13024-016-0125-0 (PMC4994244; doi:10.1186/s13024-016-0125-0)
Supplement: Additional file 2: Figure S2. — Quantification of S129 phosphorylation of human alpha-synuclein recombinant protein. An isoelectric focusing gel pH range 5–8 (A) was used to separate pS129 from non-phosphorylated h-asyn by loading recombinant h-asyn and pS129 h-asyn protein side by side. In B, blots were probed for total h-asyn (syn-1) or pS129 h-asyn (11A5) to determine pS129 h-asyn band. Quantification areas are indicated on total h-asyn blots by dashed boxes. This assay was run independently on three occasions, which yielded estimates of percent phosphorylation of h-asyn at S129 site to be 28.4, 29.5, and 26.0 %, respectively, with an average and standard deviation of 28.0 ± 1.8 %. (PDF 339 kb) [file 13024_2016_125_MOESM2_ESM.pdf]

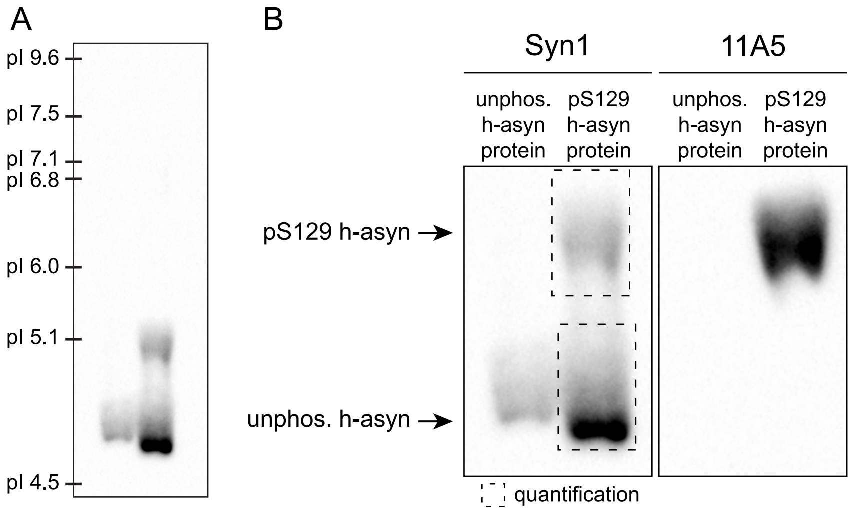

Additional file 2: Figure S2. *Quantification of S129 phosphorylation of human alpha-synuclein recombinant protein.* An isoelectric focusing gel pH range 5-8 (**A**) was used to separate pS129 from non-phosphorylated h-asyn by loading recombinant h-asyn and pS129 h-asyn protein side by side. In **B**, blots were probed for total h-asyn (syn-1) or pS129 h-asyn (11A5) to determine pS129 h-asyn band. Quantification areas are indicated on total h-asyn blots by dashed boxes. This assay was run independently on three occasions, which yielded estimates of percent phosphorylation of h-asyn at S129 site to be 28.4, 29.5, and 26.0%, respectively, with an average and standard deviation of 28.0 ± 1.8%.
