# Supplementary material for: A novel multiplex assay for simultaneous quantification of total and S129 phosphorylated human alpha-synuclein
Source: Mol Neurodegener. 2016 Aug 22;11(1):61. doi: 10.1186/s13024-016-0125-0 (PMC4994244; doi:10.1186/s13024-016-0125-0)
Supplement: Additional file 3: Figure S3. — Scheme of AlphaLISA duplex assay for total and S129 phosphorylated human alpha-synuclein detection. The 4B12 antibody – binding to aa 103–108 – is biotinylated and used to bind the Streptavidin-coated Donor-beads. Terbium Acceptor-beads were coupled to LB509 antibodies for total h-asyn quantification while the 11A5 antibody was used and attached to Europium Acceptor-beads in order to measure pS129 h-asyn. Both antibodies recognize an epitope close to each other (aa 115–122 and aa 129 respectively). (PDF 500 kb) [file 13024_2016_125_MOESM3_ESM.pdf]

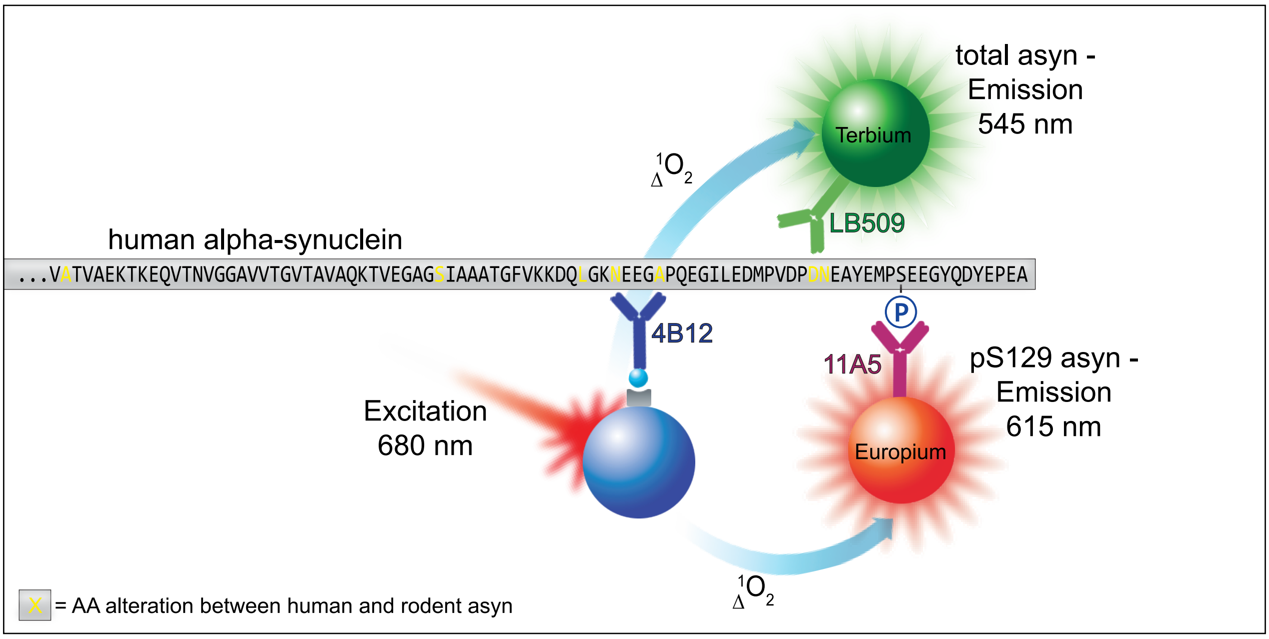

© 2009-2016 PerkinElmer, Inc. All rights reserved. Printed with permission.

Additional file 3: Figure S3. *Scheme of AlphaLISA duplex assay for total and S129 phosphorylated human alpha-synuclein detection.* The 4B12 antibody – binding to aa 103-108 – is biotinylated and used to bind the Streptavidin-coated Donor-beads. Terbium Acceptor beads were coupled to LB509 antibodies for total h-asyn quantification while the 11A5 antibody was used and attached to Europium Acceptor-beads in order to measure pS129 h-asyn. Both antibodies recognize an epitope close to each other (aa 115-122 and aa 129 respectively).
