# Supplementary material for: A novel multiplex assay for simultaneous quantification of total and S129 phosphorylated human alpha-synuclein
Source: Mol Neurodegener. 2016 Aug 22;11(1):61. doi: 10.1186/s13024-016-0125-0 (PMC4994244; doi:10.1186/s13024-016-0125-0)
Supplement: Additional file 4: Figure S4. — Principle of steric hindrance analysis and filter bleed-through. A hypothetical illustration of signal change caused by a possible steric hindrance of 11A5-Europium (11A5-Eu) and LB509-Terbium (LB509-Tb) molecules when being added together in one well is depicted in A. At baseline (A1) no steric hindrance is possible since only one Acceptor-bead coupled antibody is in solution, here shown for Terbium Acceptor-beads. Adding the second antibody (in this case 11A5) coupled to Europium Acceptor-beads can either result in signal reduction (A2) or in similar emission intensity (A3). Hypothetical signal values are illustrated in A’. Filter bleed-through for this assay protocol was determined by four measurement conditions illustrated as examples in B1-B4. First background signal for Terbium Acceptor-bead was established by using the appropriate filter as well as by not adding any analyte or Europium Acceptor-bead (B1). Background signal for Europium filter was measured by using the same well (B3). Adding the analyte to the above described mix will result in a corresponding signal when the Terbium filter is used (B2) and in a false signal (X % of Terbium signal) when the Europium filter is used (B4). Example of signals (green for Terbium, red for Europium) and % calculations is illustrated in B’. Same measurements and calculations were done for the Terbium assay protocol. (PDF 528 kb) [file 13024_2016_125_MOESM4_ESM.pdf]

 DOI 10.1186/s13024-016-0125-0

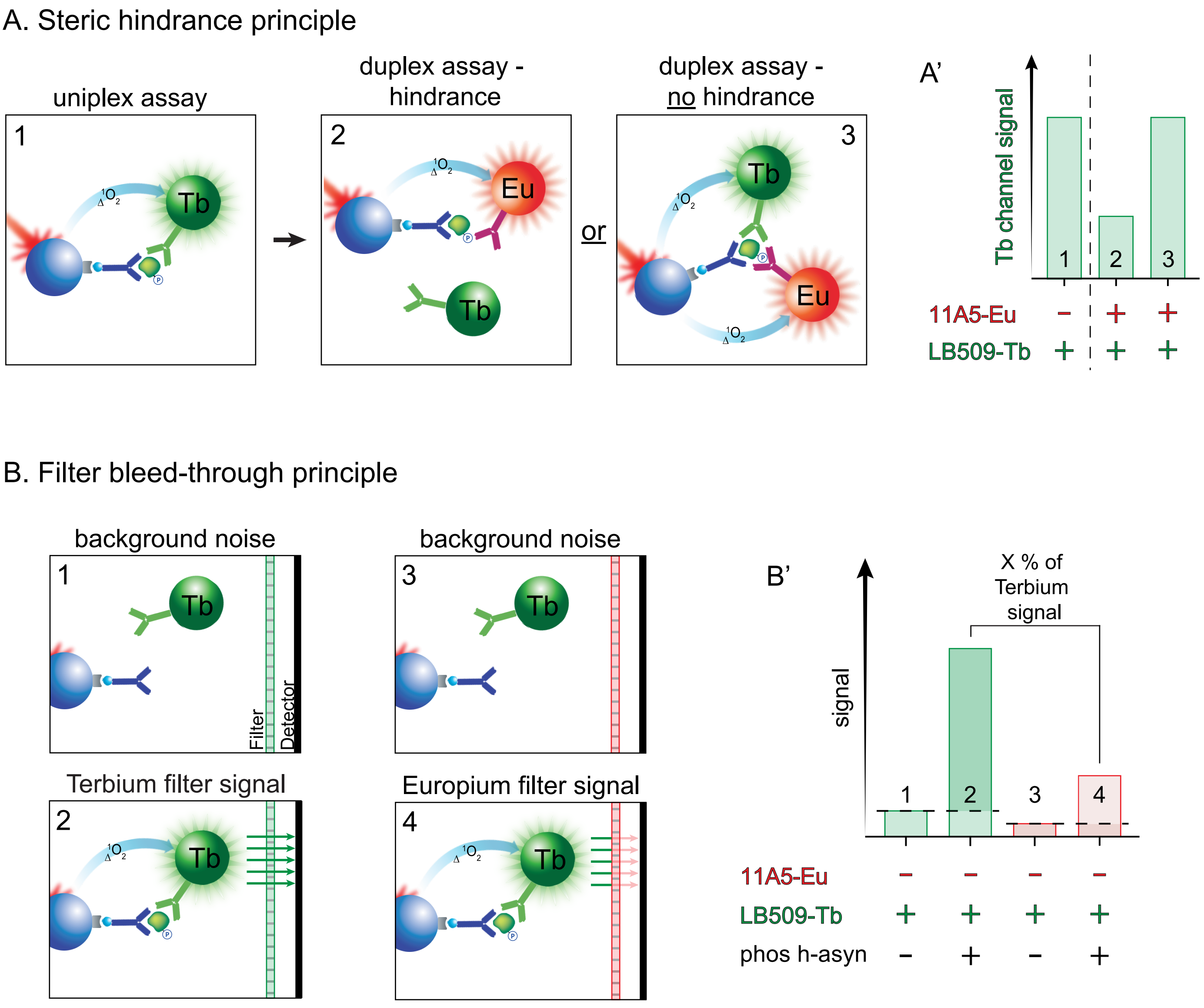

© 2009-2016 PerkinElmer, Inc. All rights reserved. Printed with permission.

Additional file 4: Figure S4. *Principle of steric hindrance analysis and filter bleed-through.* A hypothetical illustration of signal change caused by a possible steric hindrance of 11A5 Europium (11A5 Eu) and LB509-Terbium (LB509-Tb) molecules when being added together in one well is depicted in **A**. At baseline (**A1**) no steric hindrance is possible since only one Acceptor bead coupled antibody is in solution, here shown for Terbium Acceptor beads. Adding the second antibody (in this case 11A5) coupled to Europium Acceptor-beads can either result in signal reduction (**A2**) or in similar emission intensity (**A3**). Hypothetical signal values are illustrated in **A'**. Filter bleed-through for this assay protocol was determined by four measurement conditions illustrated as examples in **B1-B4**. First background signal for Terbium Acceptor-bead was established by using the appropriate filter as well as by not adding any analyte or Europium Acceptor-bead (**B1**). Background signal for Europium filter was measured by using the same well (**B3**). Adding the analyte to the above described mix will result in a corresponding signal when the Terbium filter is used (**B2**) and in a false signal (X % of Terbium signal) when the Europium filter is used (**B4**). Example of signals (green for Terbium, red for Europium) and % calculations is illustrated in B'. Same measurements and calculations were done for the Terbium assay protocol.
