# Supplementary material for: A novel multiplex assay for simultaneous quantification of total and S129 phosphorylated human alpha-synuclein
Source: Mol Neurodegener. 2016 Aug 22;11(1):61. doi: 10.1186/s13024-016-0125-0 (PMC4994244; doi:10.1186/s13024-016-0125-0)
Supplement: Additional file 5: Figure S5. — Assessment of optimal Donor-bead concentration. The ideal Donor-bead concentration was determined once simultaneously in a mix of Europium Acceptor-bead coupled 11A5 antibody and Terbium Acceptor-bead coupled LB509 antibody. Arrows indicate optimal 4B12 concentrations for both Acceptor-bead variants. AU: arbitrary units. (PDF 156 kb) [file 13024_2016_125_MOESM5_ESM.pdf]

 DOI 10.1186/s13024-016-0125-0

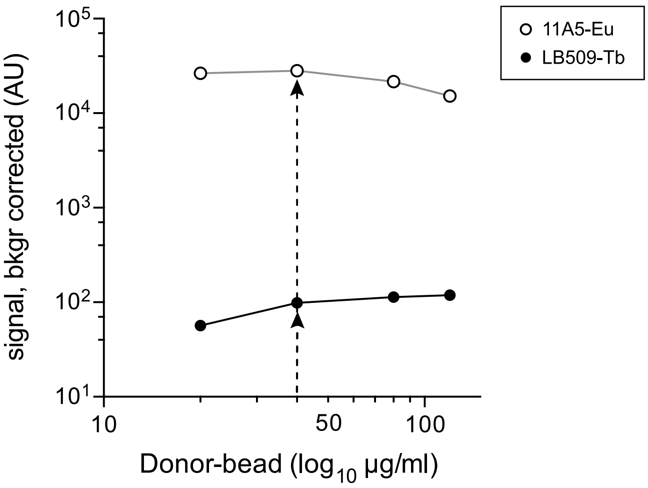

Additional file 5: Figure S5. *Assessment of optimal Donor-bead concentration.* The ideal Donor-bead concentration was determined once simultaneously in a mix of Europium Acceptor bead coupled 11A5 antibody and Terbium Acceptor-bead coupled LB509 antibody. Arrows indicate optimal 4B12 concentrations for both Acceptor-bead variants. AU: arbitrary units
