# Supplementary material for: A novel multiplex assay for simultaneous quantification of total and S129 phosphorylated human alpha-synuclein
Source: Mol Neurodegener. 2016 Aug 22;11(1):61. doi: 10.1186/s13024-016-0125-0 (PMC4994244; doi:10.1186/s13024-016-0125-0)
Supplement: Additional file 5: Figure S6. — Assessment of assay reproducibility in the duplex assay using cortical human samples. Tissue samples of the frontal cortex (n = 16) were lysed using buffer containing 1 % Triton and repeatedly analyzed (n = 5) using the duplex assay for total and pS129 h-asyn concentration on separate days. Percent variation from the mean value for each sample is plotted for total h-asyn measurements (A), for pS129 h-asyn (B) and for total to pS129 h-asyn ratio (C). Dashed lines indicate ± 25 % variation while thin lines indicate ± 10 % variation from mean (0 %). (PDF 233 kb) [file 13024_2016_125_MOESM6_ESM.pdf]

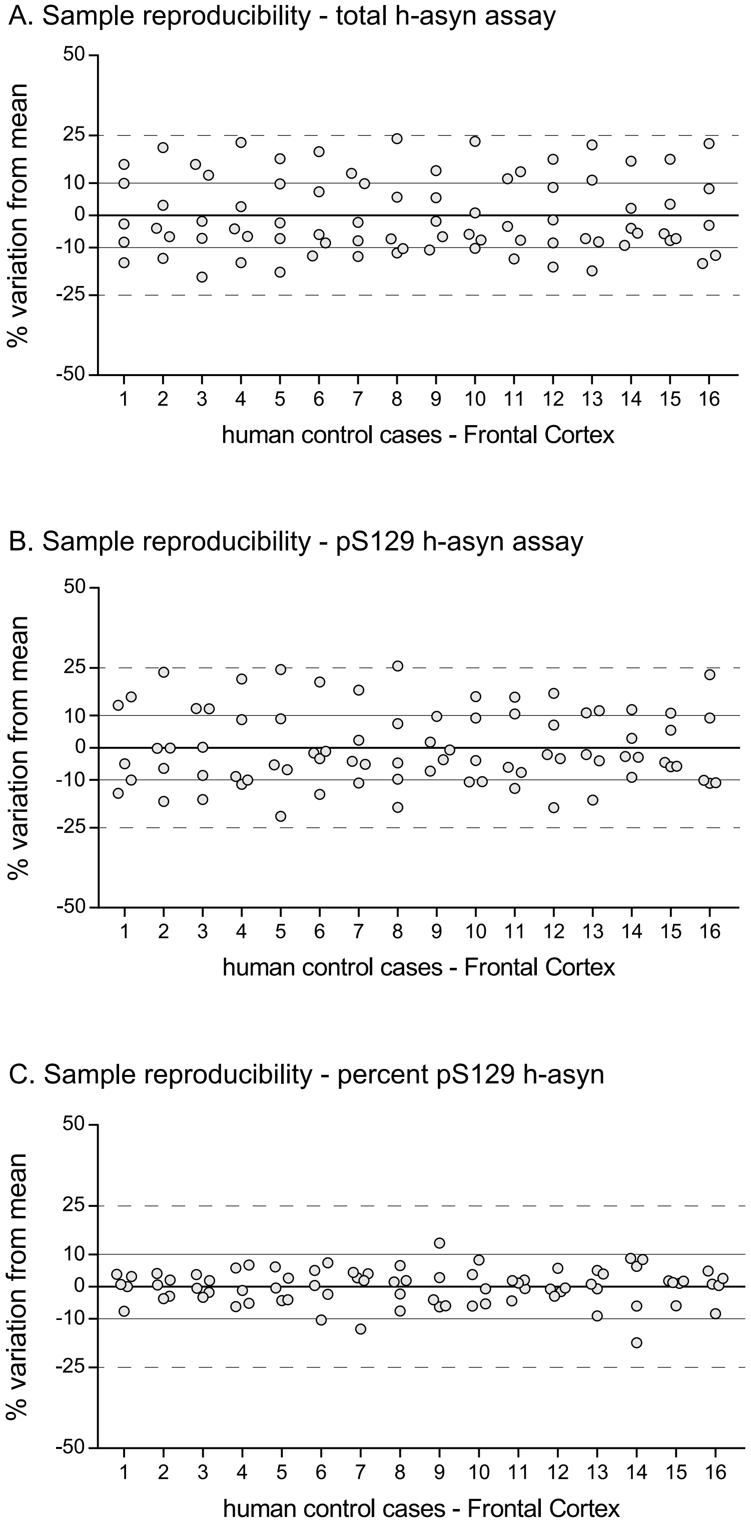

Additional file 6: Figure S6. *Assessment of assay reproducibility in the duplex assay using cortical human samples.* Tissue samples of the frontal cortex (n=16) were lysed using buffer containing 1% Triton and repeatedly analyzed (n=5) using the duplex assay for total and pS129 h-asyn concentration on separate days. Percent variation from the mean value for each sample is plotted for total h-asyn measurements (**A**), for pS129 h-asyn (**B**) and for total to pS129 h-asyn ratio (**C**). Dashed lines indicate ± 25% variation while thin lines indicate ± 10% variation from mean (0%).
