# Supplementary material for: A novel multiplex assay for simultaneous quantification of total and S129 phosphorylated human alpha-synuclein
Source: Mol Neurodegener. 2016 Aug 22;11(1):61. doi: 10.1186/s13024-016-0125-0 (PMC4994244; doi:10.1186/s13024-016-0125-0)
Supplement: Additional file 7: Figure S7. — Endogenous rat total and S129 phosphorylated levels. Fresh frozen striatal and ventral midbrain tissue samples of naïve rats were sequentially lysed in first 1 % Triton (A) then in 1 % SDS containing lysis buffer (B) and diluted 1:300 or 1:10, respectively. Samples (n = 6 per region) were run on AlphaLISA uniplex assays using the biotinylated syn-1 antibody (BD Biosciences, UK) combined with either 4D6 (Abcam, UK) or 11A5 (Prothena Biosciences Inc.) conjugated on the Europium Acceptor-beads for the detection of total and pS129 asyn, respectively. As reference protein, pS129 h-asyn was used for both assays and spiked into appropriate asyn knockout mouse brain lysate. Total (open) and pS129 (filled) r-asyn levels are presented in overlapping bars. Numbers inside the bars show the percent of pS129 to total r-asyn. Error bars indicate SEM. (PDF 173 kb) [file 13024_2016_125_MOESM7_ESM.pdf]

 DOI 10.1186/s13024-016-0125-0

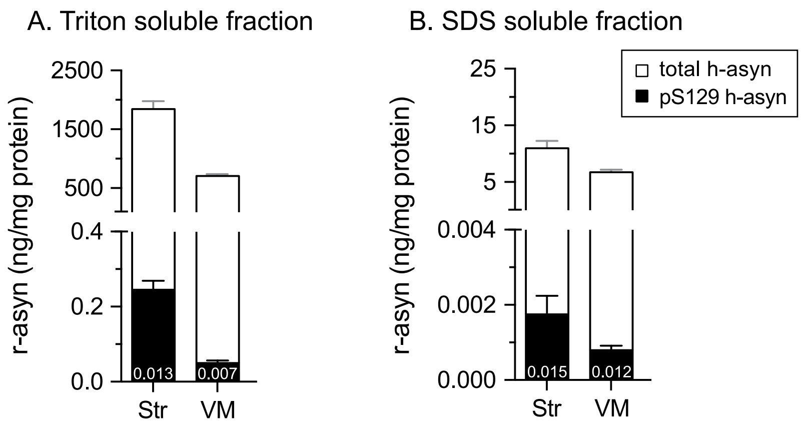

Additional file 7: Figure S7. *Endogenous rat total and S129 phosphorylated levels.* Fresh frozen striatal and ventral midbrain tissue samples of naïve rats were sequentially lysed in first 1% Triton (**A**) then in 1% SDS containing lysis buffer (**B**) and diluted 1:300 or 1:10, respectively. Samples (n=6 per region) were run on AlphaLISA uniplex assays using the biotinylated syn-1 antibody (BD Biosciences, UK) combined with either 4D6 (Abcam, UK) or 11A5 (Prothena Biosciences Inc.) conjugated on the Europium Acceptor-beads for the detection of total and pS129 asyn, respectively. As reference protein, pS129 h-asyn was used for both assays and spiked into appropriate asyn knockout mouse brain lysate. Total (open) and pS129 (filled) r-asyn levels are presented in overlapping bars. Numbers inside the bars show the percent of pS129 to total r-asyn. Error bars indicate SEM.
